# Identification of the Molecular Events Involved in the Development of Prefrontal Cortex Through the Analysis of RNA-Seq Data From BrainSpan

**DOI:** 10.1177/1759091419854627

**Published:** 2019-06-18

**Authors:** Hadi Najafi, Mohadeseh Naseri, Javad Zahiri, Mehdi Totonchi, Majid Sadeghizadeh

**Affiliations:** 1 Department of Genetics, Faculty of Biological Sciences, Tarbiat Modares University, Tehran, Iran; 2 Department of Biophysics, Bioinformatics and Computational Omics Lab (BioCOOL), Faculty of Biological Sciences, Tarbiat Modares University, Tehran, Iran; 3 Department of Genetics and Stem Cell, Royan Institute, Tehran, Iran

**Keywords:** bioinformatics, BrainSpan, gene set enrichment analysis, global transcription rate, prefrontal cortex

## Abstract

Human brain development is a complex process that follows sequential orchestration of gene expression, begins at conceptual stages, and continues into adulthood. Altered profile of gene expression drives many cellular and molecular events required for development. Here, the molecular events during development of human prefrontal cortex (PFC) (as an important executive part of the brain) were investigated. First, the RNA-sequencing data of BrainSpan were used to obtain differentially expressed genes between each two developmental stages and then, the relevant biological processes and signaling pathways were deduced by gene set enrichment analysis. In addition, the changes in transcriptome landscape of PFC during development were analyzed and the potential biological processes underlie the changes were found. Comparison of the four regions of PFC based on their biological processes showed that additional to common biological processes and signaling pathways, each PFC region had its own molecular characteristics, conforming their previously reported functional roles in brain physiology. The most heterogeneity in transcriptome between the PFC regions was observed at the time of birth which was concurrent with the activity of some region-specific regulatory systems such as DNA methylation, transcription regulation, RNA splicing, and presence of different transcription factors and microRNAs. In conclusion, this study used bioinformatics to present a comprehensive molecular overview on PFC development which may explain the etiology of brain neuropsychiatric disorders originated from malfunctioning of PFC.

## Introduction

Brain development is a very complex, dynamic, and multistage process that involves precisely orchestrated sequence of cellular and molecular events (Silbereis et al., 2016). This development begins within weeks of conception (∼third gestational week) and continues through the adolescence (Stiles and Jernigan, 2010). Many controlled morphogenesis processes such as cell division, differentiation, migration, and neural fate specifications generate distinct parts that are cooperatively functional in architecture of the brain (Anderson et al., 2013). Of all brain regions, the prefrontal cortex (PFC) (the anterior part of the frontal lobe) coordinates a wide range of cognitive processes whose cardinal role is mediating complex behaviors rather than basic cognitive activities (Frith and Dolan, 1996). Malfunctioning of the PFC is greatly linked to many human cognitive problems (Siddiqui et al., 2008) as well as psychiatric disorders such as bipolar disorder (Clark and Sahakian, 2008; Abé et al., 2015), schizophrenia (Wible et al., 2001), attention-deficit or hyperactivity disorder (ADHD) (Vaidya, 2011), drug addiction (Goldstein and Volkow, 2011), autism (Goldberg et al., 2011), and depression (Koenigs and Grafman, 2009). Thus, understanding the details of molecular events that occur during maturation of this part may be an important issue for resolving various dilemmas concerning such phenotypes. PFC comprises four distinct parts including dorsolateral prefrontal cortex (DFC), medial prefrontal cortex (MFC), ventrolateral prefrontal cortex (VFC), and orbital frontal cortex (OFC) which have subtle differences in their physiology and tasks during human lifetime (Yu et al., 2016). Although there is a functional connectivity throughout PFC and also throughout whole brain (Pessoa, 2014), most studies surveyed the physical and functional changes during maturation of PFC and there is little known about the genetic and molecular changes across the developing human brain. Since, expression alteration in a subset of genes may be critical to drive one premature developmental stage to the next (Erraji-Benchekroun et al., 2005), it is of great importance to find and analyze the genes responsible for each developmental stages. Interestingly, BrainSpan database is the atlas of the developing human brain providing a detailed global characterization and comparison of the genes separately expressed by 26 different brain regions during 12 developmental times. This article exploits the RNA-seq data of this database to depict the major molecular events occurred during development of PFC from its early embryonic state through adolescence.

## Materials and Methods

### Study Design

**Figure 1.**
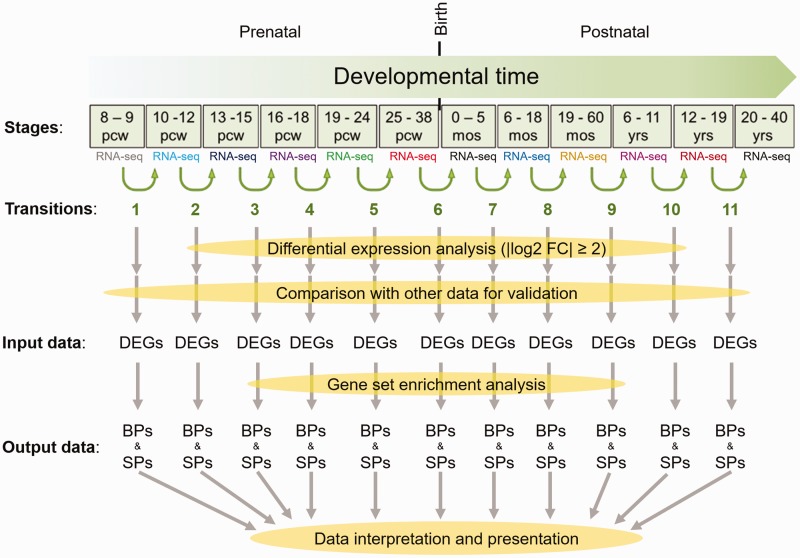
Workflow of this study from data collection to processing steps. Based on the RNA-seq data of 12 developmental stages of developing human brain, DEGs between two immediate stages were identified. After validation of the identified DEGs through their comparison with microarray data, they were used as input for gene set enrichment analysis by DAVID. Finally, analysis of the BPs and SPs, for these DEGs was performed. Output data of the workflow (BPs and SPs) were further interpreted and illustrated in next analytic steps of this study. pcw = postconceptual weeks; mos = months after birth; yrs = years after birth; DEG = differentially expressed genes; BP = biological processes; SP = signaling pathways.

In spite of the existence of numerous reports on functional role of many genes in brain development, there is a lack of a comprehensive study to describe molecular events across developmental process of human brain, from conceptual stages to adulthood. Therefore, this study was designed to identify potential biological processes and signaling pathways, responsible for development of human PFC as a prominent executive part of the brain. To do so, gene expression profile (RNA-seq data) of one developmental stage of PFC was compared with the next stage, and the resultant differentially expressed genes (DEGs) were analyzed to obtain relevant biological processes and signaling pathways. Thereafter, these biological processes or signaling pathways were interpreted and their relevance to different times of PFC development was described (Figure 1). In addition, for validation or comparison of the obtained results, similar approaches were performed on the raw (RNA-seq and microarray) data of BrainSpan as well as analyzing the microarray data produced by Kang et al. (2011) (GEO number: GSE25219).

### Differentially Expressed Genes

The RNA-seq and microarray data of different parts of human brain for 12 different developmental stages are deposited in the Allen Institute for Brain Science BrainSpan Atlas (http://www.brainspan.org), and the details of tissue acquisition, processing, and RNA-sequencing can be found on the website.

In this study, the RNA-seq and microarray data of four distinct regions of PFC across different times of brain development were used. These four regions include DFC, MFC, VFC, and orbital frontal cortex (OFC). The DEGs between two immediate developmental stages of each region were obtained by “differential search” approach of the BrainSpan database (http://www.brainspan.org/rnaseq/search/index.html). This website compares the expression profile of a developmental stage with another stage and represents the genes with absolute log_2_ fold change (FC).

Additional to the online analysis by BrainSpan, the raw datasets of BrainSpan and also the microarray data of Kang et al. (GSE25219) were downloaded and analyzed by the R package limma (Smyth et al., 2005) for validation of our DEGs. Prior to comparison of developmental stages and in order to ruling out the effects of heterogeneity in sample sizes of developmental stages, “downsampling” approach was performed. For this purpose, two samples (one male and one female) of the existed samples in each developmental stage were selected randomly. Thereafter, for validation of the results, the data of downsampling and those of total sampling were compared with each other, using Pearson correlation coefficient analysis. Using Venn diagram by the online tool Bioinformatics and Evolutionary Genomics (http://bioinformatics.psb.ugent.be/webtools/Venn/), we found the overlapped data between the DEGs resulted from RNA-seq data analysis and those of microarray data sources.

For both RNA-seq and microarray data, genes were considered as significantly differentially expressed between groups when false discovery rate *p* was <.05 and the absolute log_2_ (FC) was ≥2.

### Functional Classification of DEGs

To identify which biological processes and signaling pathways are involved in the development of PFC, gene ontology (GO) analysis of biological processes and signaling pathways was performed by the “Database for Annotation, Visualization and Integrated Discovery” (DAVID) (https://david.ncifcrf.gov/). The lists of DEGs (Supplemental File S1) were used as input of the DAVID software, and the results were presented based on GO terms and Kyoto Encyclopedia of Genes and Genomes (KEGG) pathways. Adjusted *p* < .05 was considered as statistically significant of the enrichments.

### Gene Coexpression Analysis

We used the R package weighted gene coexpression network analysis (WGCNA) (Zhang and Horvath, 2005; Langfelder and Horvath, 2008) for analysis of the raw RNA-seq data of BrainSpan to find modules of coexpressed genes, potentially involved in the development of PFC. In detail, first, gene clustering tree was created based on expression similarity of the genes across all developmental stages of PFC. Following pruning the less connected nodes, the modules with the maximal number of the genes (more than 600 genes) were retained for subsequent analysis. To determine PFC-specificity of the modules, we used Jaccard index to compare the modules with WGCNA data of other brain regions. To summarize the expression profile of each module, principle component analysis (PCA) was performed. The resulting PC1 was plotted against developmental stages, and a smooth curve was fitted by smoothing spline to display the developmental trajectories of the modules.

### Gene Expression Trajectories

To investigate cell type switching during PFC development, we utilized the raw RNA-seq data of BrainSpan and explored the expression pattern of some selected genes which previously reported as specific markers of different cell types of central nervous system (such as Astrocytes, Oligodendrocytes, microglia, neurons and neural stem/progenitor cells) (Cahoy et al., 2008; Artegiani et al., 2017; McKenzie et al., 2018).

The same procedure was also performed for three general transcription factors (TFs) (TBP, RNase-H1 and SPARCA2) which were previously reported as the genes tightly relevant to RNA polymerase activity (Kotsantis et al., 2016). Expression level of the genes was calculated as RPKM.

### Global Transcription Rate Assessment

To acquire a view on the role of global transcription regulation (RNA polymerase activity level) in development of PFC, relevant GO terms of biological processes in each developmental stage (Supplemental File S2) were taken into account (Table 1). Accordingly, the value of “global transcription rate” was calculated by the sum of all positive and negative regulatory processes of DEGs for each developmental transition.

**Table 1.** The GO Terms of Biological Processes Which Were Summed to Calculate the Global Transcription Rate Throughout Development of Human Prefrontal Cortex.

No.	GO terms	Effects
1	Negative regulation of transcription regulatory region DNA binding	−
2	Negative regulation of transcription, DNA-templated	−
3	Negative regulation of transcription from RNA polymerase II promoter	−
4	Transcription initiation from RNA polymerase II promoter	+
5	Transcription from RNA polymerase II promoter	+
6	Positive regulation of transcription, DNA-templated	+
7	Positive regulation of sequence-specific DNA binding transcription factor activity	+
8	Positive regulation of transcription from RNA polymerase II promoter	+
9	Transcription, DNA-templated	+
10	Positive regulation of transcription of nuclear large rRNA transcript from RNA polymerase I promoter	+
11	mRNA transcription from RNA polymerase II promoter	+
12	Positive regulation of pri-miRNA transcription from RNA polymerase II promoter	+

*Note.* “+” and “−,” respectively, denote positive and negative regulatory effects of these processes on transcription rate. Except the biological processes in rows 1 to 3 (with “−” signs), other biological processes (with “+” signs) are positive regulators of transcription. GO = gene ontology.

### Principle Component Analysis

We performed PCA on the raw RNA-seq data of the BrainSpan to compare the transcriptome of the four studied regions of PFC (DFC, MFC, VFC and OFC).

### Statistical Analysis

The statistical analysis tools Excel and GraphPad Prism (version 6) were used for analysis and representation of data. The value of *p* less than .05 was considered as statistically significant. Correlation analysis was also performed using GraphPad Prism (version 6) software, represented as a Pearson coefficient. Regression and *x*-bar analysis were performed by Minitab (version 16) software.

## Results

### Identification of Potential Biological Processes Involved in the Development of Human PFC

PFC is known to be divided into four regions (DFC, MFC, VFC, and OFC) whose development may require activity of many biological processes. Therefore, using RNA-seq data of 12 different developmental stages of these regions, DEGs between each of the two subsequent stages were initially derived from BrainSpan (Figure 1). Thereafter, gene set enrichment analysis of these DEGs by DAVID gene classifier predicted many biological processes that are potentially important for transition from one developmental stage to the next (Supplemental File S2). In addition, for validation of these DEGs, they were compared with the DEGs obtained from the microarray data of BrainSpan and Kang et al. (GEO number: GSE25219).

**Figure 2.**
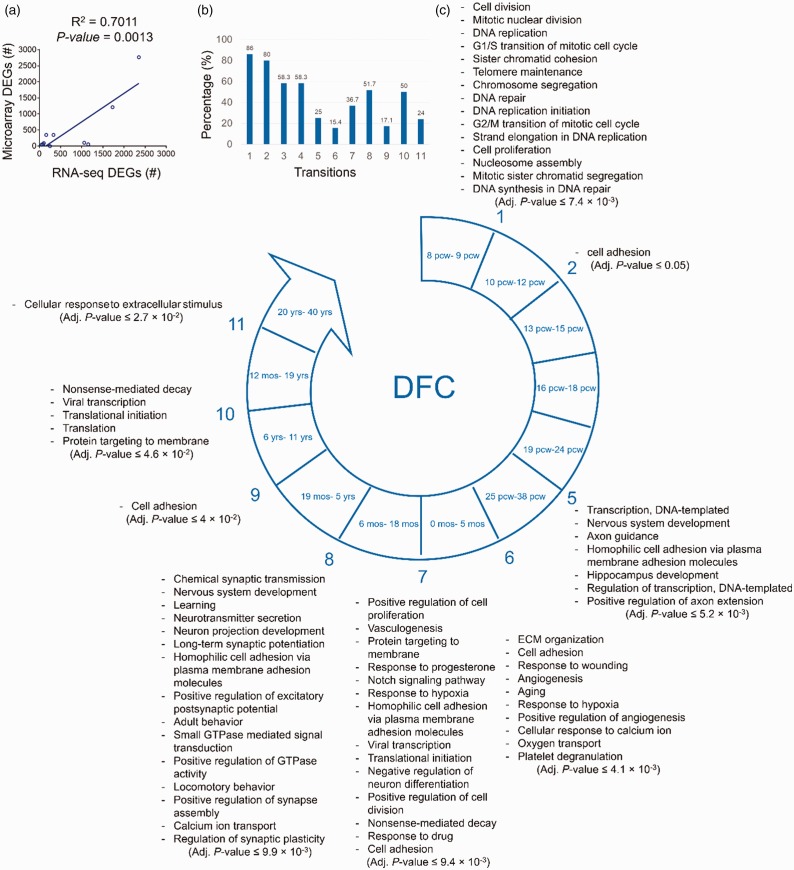
The biological processes predicted to be involved in each developmental transition of DFC. (a) The significant positive correlation between the number of the DEGs identified by analysis of microarray (*y*-axis) and RNA-seq (*x*-axis) data. (b) The degree of similarity between the DEGs identified through microarray data analysis and those from RNA-seq data analysis. Bars represent the percentages of the microarray DEGs which also were identified by RNA-seq data as DEGs. (c) The enriched biological processes predicted to be involved in different developmental transitions of DFC. Adjusted *p* < .05 was considered as statistically significant. Numbers show developmental transitions with their corresponding biological processes. The transitions without numbers had no significant enriched biological processes. DFC = dorsolateral prefrontal cortex; DEG = differentially expressed genes.

**Figure 3.**
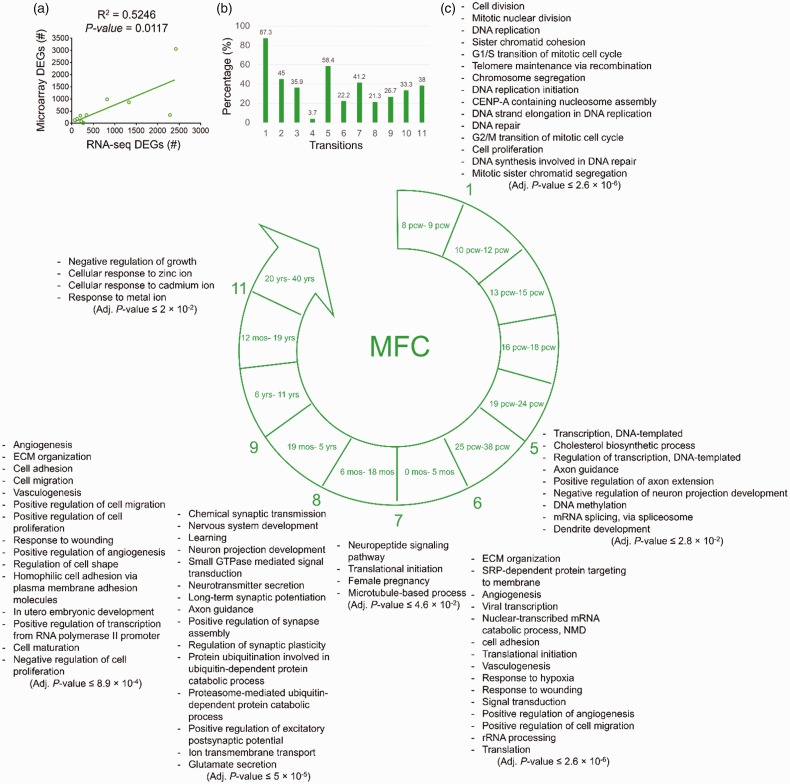
The biological processes predicted to be involved in each developmental transition of MFC. (a) The significant positive correlation between the numbers of the DEGs identified by analysis of microarray (y-axis) and RNA-seq (*x*-axis) data. (b) The degree of similarity between the DEGs identified by microarray data analysis and those from RNA-seq data analysis. Bars represent the percentages of the microarray DEGs which were also identified as DEGs by RNA-seq data analysis. (c) The enriched biological processes predicted to be involved in different developmental transitions of MFC. Adjusted *p* < .05 was considered as statistically significant. Numbers show developmental transitions with their corresponding biological processes. The transitions without numbers had no significant enriched biological processes. MFC = medial prefrontal cortex; ECM = extracellular matrix; SRP = signal recognition particle; NMD = nonsense-mediated decay; CENP = centromere protein.

**Figure 4.**
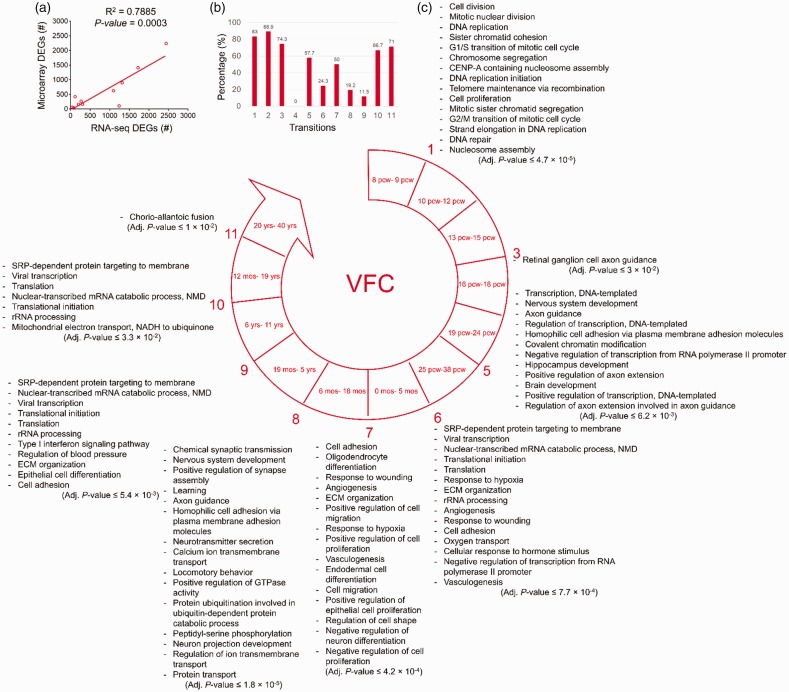
The biological processes predicted to be involved in each developmental transition of VFC. (a) The significant positive correlation between the numbers of the DEGs identified by analysis of microarray (*y*-axis) and RNA-seq (*x*-axis) data. (b) The degree of similarity between the DEGs identified by microarray data analysis and those from RNA-seq data analysis. Bars represent the percentages of the microarray DEGs which were also identified as DEGs by RNA-seq data analysis. (c) The enriched biological processes predicted to be involved in different developmental transitions of VFC. Adjusted *p* < .05 was considered as statistically significant. Numbers show developmental transitions with their corresponding biological processes. The transitions without numbers had no significant enriched biological processes. ECM = extracellular matrix; SRP = signal recognition particle; NMD = nonsense-mediated decay; VFC = ventrolateral prefrontal cortex; CENP = centromere protein.

**Figure 5.**
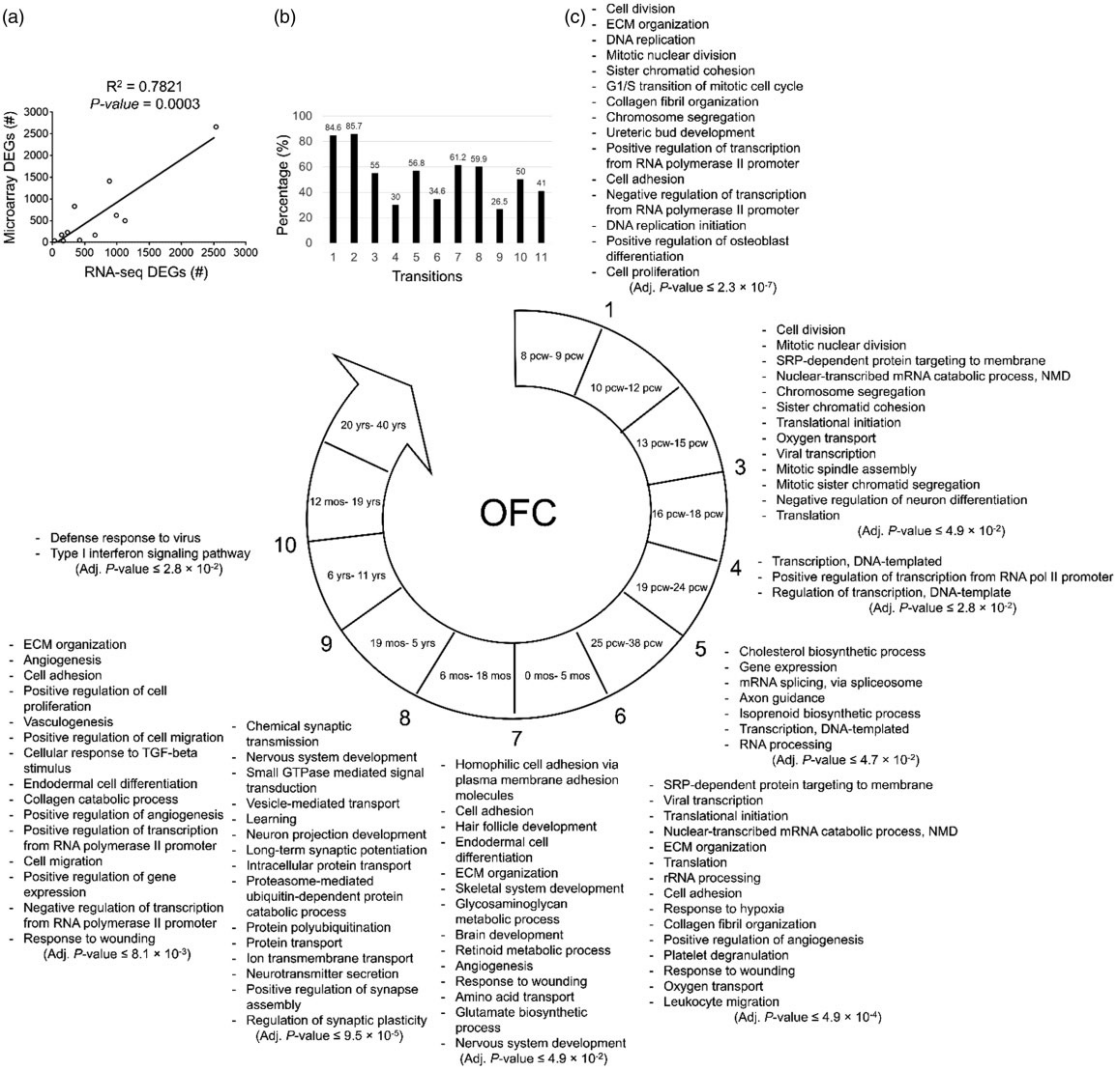
The biological processes predicted to be involved in each developmental transition of OFC. (a) The significant positive correlation between the numbers of the DEGs identified by analysis of microarray (*y*-axis) and RNA-seq (*x*-axis) data. (b) The degree of similarity between the DEGs identified by microarray data analysis and those from RNA-seq data analysis. Bars represent the percentages of the microarray DEGs which were also identified as DEGs by RNA-seq data analysis. (c) The enriched biological processes predicted to be involved in different developmental transitions of OFC. Adjusted *p* < .05 was considered as statistically significant. Numbers show developmental transitions with their corresponding biological processes. The transitions without numbers had no significant enriched biological processes. OFC = orbital frontal cortex; TGF = transforming growth factor; ECM = extracellular matrix; NMD = nonsense-mediated decay; DEG = differentially expressed genes.

Using the RNA-seq DEGs as the input of DAVID software, the results of gene enrichment analysis for all the studied developmental stages of PFC regions together with their corresponding biological processes were presented (Figures 2 to 5). For such enrichment analysis of biological processes, both level of expression changes (|Log_2_ FC| ≥ 2) and *p* value (Adjusted *p* < .05) were considered as the threshold of statistical significance.

The results of comparison of RNA-seq and microarray data for DFC region showed a significant positive correlation (*R*^2^ = .711, *p* value = .0013) between their numbers of DEGs (Figure 2(a)). Also, most of the microarray DEGs were overlapped with the analyzed RNA-seq DEGs (Figure 2(b)).

Enrichment analysis of the DEGs for DFC showed that the biological processes linked to cell division, DNA replication, telomere maintenance, and DNA repair were significantly enriched for the Transition 1. The biological process of cell division is continued to the Transition 2, while the Transitions 3 and 4 had no significantly enriched biological processes.

During Transition 5, biological processes of transcription regulation, nervous system development, hippocampus (HIP) development, cell adhesion, and axon guidance or extension were enriched. During developmental Transition 6, extracellular matrix (ECM) organization, cell adhesion, angiogenesis, oxygen transport, aging, response to calcium, and responses to stresses (such as wounding and hypoxia) were significantly enriched.

Concerning Transition 7 of the DFC development, the biological processes of cell proliferation, cell adhesion, neuronal differentiation, and responses to progesterone, drugs, and hypoxia were enriched. Other processes of this transition were translation initiation, nonsense-mediated decay (NMD), protein targeting to membrane, and notch signaling pathway.

The Transition 8 harbors the biological processes related to synaptic development (chemical synaptic transmission, neurotransmitter secretion, neuron projection, synaptic potentiation, synaptic assembly, and plasticity), nervous system development, learning, behavior, and GTPase-mediated pathways.

In Transition 9, cell adhesion was significantly enriched. The biological processes of NMD, protein translation, and protein targeting to membrane were significantly enriched. In the last developmental transition of DFC, the biological process of cellular response to extracellular stimuli was enriched.

The developmental stages (transitions) of DFC with their enriched biological processes are shown in Figure 2(c).

The results of the comparison between RNA-seq and microarray data of MFC region showed a significant positive correlation (*R*^2^ = .5246, *p* = .0117) between their numbers of DEGs (Figure 3(a)). Also, the overlap between the lists of microarray DEGs and the analyzed lists of RNA-seq DEGs is shown in Figure 3(b).

Like DFC, the first developmental transition of MFC was concurrent with the biological processes related to cell division, DNA replication, telomere maintenance, and DNA repair.

There was no prediction of any biological processes for the DEGs of the Transitions 2, 3, and 4 in MFC. The Transition 5 of MFC was concurrent with the biological processes of Transcription regulation, cholesterol biosynthesis, development of axons and dendrites, DNA methylation, and RNA splicing.

During Transition 6 of MFC, ECM organization, cell adhesion, cell migration, NMD, rRNA processing, protein translation, protein targeting to membrane, angiogenesis, responses to wounding, and hypoxia were significantly enriched. The enriched biological processes of Transition 7 in MFC were neuropeptide signaling pathway, translational initiation, female pregnancy, and microtubule-based processes.

Most of the enriched biological processes in Transition 8 of MFC were similar to those of DFC, except the biological process of protein degradation via ubiquitination and proteasome.

During Transition 9 of MFC, the biological processes related to ECM organization, cell proliferation, cell migration, cell adhesion, regulation of cell shape, cell maturation, and angiogenesis were significantly enriched. While no significant biological process was observed for the Transition 10, in the Transition 11 of MFC, the enriched biological processes were negative regulation of growth and cellular responses to metal ions (including zinc and cadmium) (Figure 3(c)).

Comparing the number of microarray DEGs and that of RNA-seq DEGs showed a significant positive correlation in VFC (*R*^2^ = .7885, *p* = .0003) which could confirm the DEGs identified by RNA-seq data of the BrainSpan (Figure 4(a)). Comparing the lists of the DEGs from microarray and those of the RNA-seq data also showed an overlap between the DEGs within each developmental transition (Figure 4(b)).

In VFC, like aforementioned regions, the enriched biological processes of the first developmental transition were related to cell division, DNA replication, telomere maintenance, and DNA repair. While no significant biological processes were predicted for DEGs of the Transitions 2 and 4 of MFC, the biological process of “retinal ganglion cell axon guidance” was significantly enriched for Transition 3 of VFC.

The enriched biological processes for Transition 5 of VFC were transcription regulation, covalent chromatin modification, nervous system and brain development, and axon development (axon extension and guidance).

The biological processes of transcription regulation, rRNA processing, NMD, protein translation, protein targeting to membrane, cellular responses (to hypoxia and wounding), angiogenesis, and ECM organization were significantly enriched for Transition 6 of VFC.

The Transition 7 of VFC is concurrent with differentiation of oligodendrocytes, neurons, and endodermal and epithelial cells. Other enriched biological processes are cell adhesion, ECM organization, cell proliferation and migration, responses (to wounding and hypoxia), and angiogenesis.

The biological processes in Transition 8 of VFC were related to nervous system development, synaptic development (chemical synaptic transmission, neurotransmitter secretion, neuron projection, synaptic potentiation, synaptic assembly, and plasticity), ion transmembrane transport (calcium), cell adhesion, learning, behavior, GTPase-mediated pathways, and protein degradation via ubiquitination and proteasome.

During Transition 9 of VFC, in addition to the biological processes of NMD, rRNA processing, translation initiation, protein targeting to membrane, ECM organization, cell adhesion, epithelial cell differentiation, the biological processes of “Type-1 interferon signaling pathway,” and “regulation of blood pressure” were enriched.

In Transition 10, the biological processes of rRNA processing, NMD, protein translation, and protein targeting to membrane were enriched in addition to the emergence of mitochondrial electron transport, NADH to ubiquinone.

In the last transition of VFC, the biological process of “chorioallantoic fusion” was significantly enriched (Figure 4(c)).

In OFC, in order to validate the DEGs of BrainSpan RNA-seq, they were compared with the lists of the DEGs of microarray data. Results showed a positive correlation (*R*^2^ = .7821, *p* = .0003) between the number of RNA-seq and microarray DEGs (Figure 5(a)) and also there were overlaps between these two types of DEGs (Figure 5(b)).

The first developmental transition of OFC is enriched for the biological processes of cell division, DNA replication, DNA repair, transcription from RNA polymerase II, collagen fibril organization, uretic bud development, and osteoblast differentiation where the latest four biological processes are unique to this region (see Figures 2 to 5; Transition 1).

While there was no significant enrichment for the Transition 2 of OFC, the biological processes of mitotic cell division, chromatin dynamics (aggregation and segregation), NMD protein translation, protein targeting to membrane, oxygen transport, and negative regulation of neuron differentiation were significantly enriched for Transition 3. Transition 4 of OFC is concurrent with biological processes related to transcription regulation.

The enriched biological processes for Transition 5 of OFC were regulation of gene expression, RNA processing and mRNA splicing, cholesterol biosynthesis, isoprenoid biosynthesis, and axon guidance. For the developmental Transition 6 of OFC, the biological processes of rRNA processing, NMD, protein translation, protein targeting to membrane, ECM organization, angiogenesis, responses (to hypoxia and wounding), platelet degranulation, and leukocyte migration. The two later processes may mediate the activation of immune system in this transition. The enriched biological processes of the Transition 7 of OFC were related to cell adhesion, hair follicle development, endodermal cell differentiation, ECM organization, skeletal system development, nervous system and brain development, angiogenesis, response to wounding, amino acid transport and metabolic processes of glycosaminoglycans (GAGs), retinoid, and glutamate biosynthesis.

During Transition 8, the biological processes of synaptic development and activity, nervous system development, intracellular protein transport, vesicle-mediated transport, neurotransmitter secretion, ion transportation, protein degradation via ubiquitination and proteasome, and GTPase-mediated signaling were enriched. The Transition 9 of OFC was predicted to be linked with the biological processes of ECM organization, cell adhesion, cell proliferation, angiogenesis, cell migration, endodermal cell differentiation, responses to wounding and TGF-β signaling, and regulation of transcription by RNA polymerase II. The transition of 10 of OFC is concurrent with two immune system-related processes: defense response to virus and Type-1 interferon signaling pathway. However, there was no enriched biological process for the last transition of the OFC region (Figure 5(c)).

### Orchestrated Change of the Involved Biological Processes in the Development of PFC

**Figure 6.**
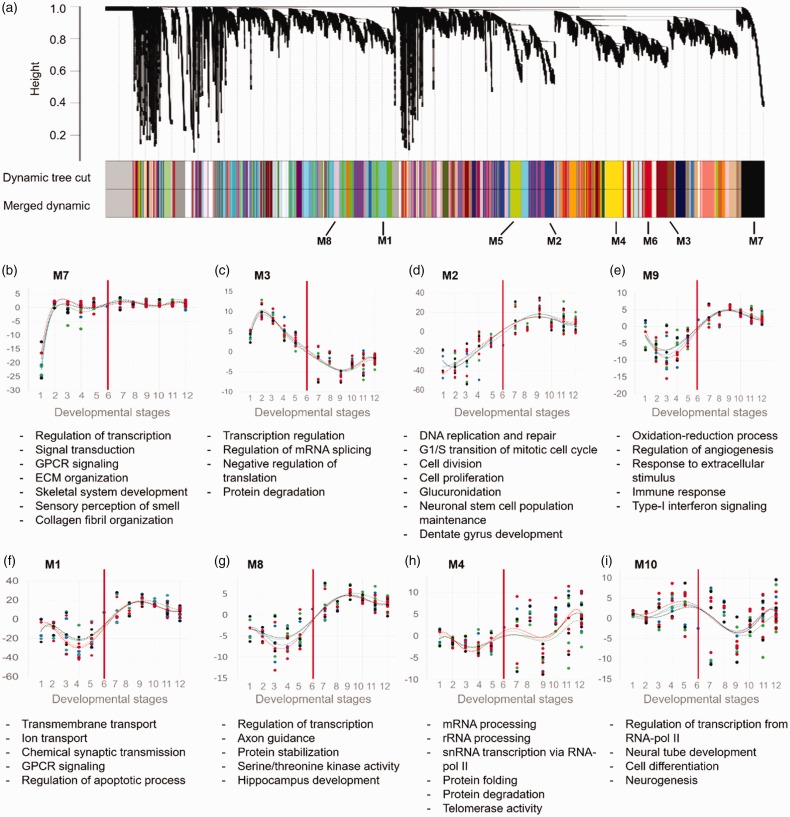
Gene clusters and their corresponding biological processes involved in the development of prefrontal cortex. (a) Clustering result of WGCNA on transcriptome of the prefrontal cortex. Modules of coexpressed genes are shown as colored box below the dendrogram. Eight modules (M1–M8) showed at least 600 genes with common expression trend during development. (b–i) The trajectory of each modules together with their enriched biological processes, predicted by DAVID software. For all the enrichment analysis, an adjusted *p* value < .05 was considered as statistically significant. Vertical red lines denote the time of birth. GPCR = G-protein coupled receptor; ECM = extracellular matrix.

To find the modules of coexpressed genes across development of PFC, the R package WGCNA was used. A total number of 61 modules were predicted for PFC (Figure 6(a)) which were not observed in WGCNA results of other brain regions (Jaccard index < 0.0037; Supplemental File S3). We also investigated trajectories of the modules across development of PFC by calculating the module eigengene. Thereafter, biological processes related to each module were predicted by DAVID software.

Results showed the earliest change for Module 7 (M7) which started from the Stage 1 (8–9 postconceptual weeks [pcw]) of prefrontal development. Predicted biological processes for this module were transcription regulation, signal transduction, signaling by G-protein-coupled receptors, ECM organization, skeletal system development, and collagen fibril organization. The activity level of this module is sustained until adulthood, suggesting the importance of its genes in all developmental stages (Figure 6(b)).

The second increase in activity was observed for the Module 3 (M3) which was enriched for transcription regulation, mRNA splicing, negative regulation of translation, and protein degradation occurring at Stage 2 (10–12 pcw). However, this module has a decreasing expression pattern as the PFC is developing (Figure 6(c)).

The next upregulated module was M2 which was increased at Stage 3 (13–15 pcw). The genes in this module mainly contribute in DNA replication, DNA repair, cell cycle promotion, cell proliferation, neuronal stem cell maintenance, and dentate gyrus development. This module gradually is increased during PFC development until the Stage 9 (19–60 months after birth). After this time, the level of this module is remained in a steady state (Figure 6(d)).

In the postnatal stages of PFC development, upregulation of the modules M1, M8, and M9 was observed. The corresponding biological processes include oxidation–reduction processes, angiogenesis, and response to stimuli and immune system (for M9); transmembrane transportation of ions, chemical synaptic transmission, G-protein-coupled receptors signaling, and regulation of apoptosis (for M1); and regulation of transcription, axon guidance, protein stabilization, serine or threonine kinase activity, and HIP development (for M8). The lowest expressions of these modules were observed in prenatal stages (13–18 pcw) (Figure 6(e) to (g)).

Unlike stage-specific modules (biological processes), there were two modules (M4 and M10) which exhibited ever-changing patterns in their expression which may highlight their importance throughout the development. Module 4 (M4) modules were predicted to be involved in gene expression regulation (e.g., mRNA splicing, rRNA processing, and transcription of snRNAs by RNA-pol II), telomerase activity, and protein metabolism (Figure 6(h)). For Module 10 (M10), the biological processes of neural tube development, cell differentiation, and neurogenesis were enriched, suggesting the active neural system development throughout PFC development (Figure 6(i)). Taken together, this result shows that PFC development is mediated by sequential expression alterations of the genes which drive several biological processes.

### Cell Type Conversions During Development of PFC

**Figure 7.**
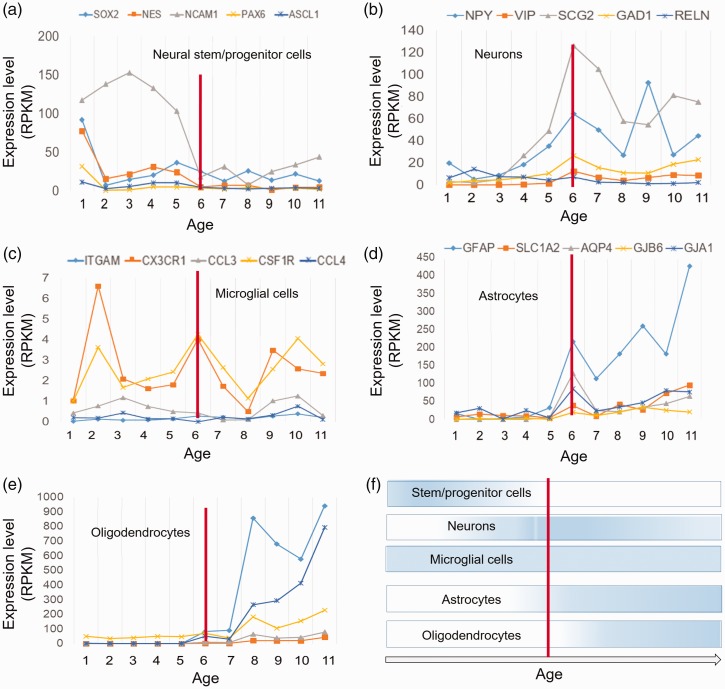
Trajectory of different neural cell markers during development of prefrontal cortex. (a–d) Trajectories of the five marker genes or each cellular type of neural system. Gene names are presented on the top of each graph with color codes. Expression levels are presented as RPKM. (e) A schematic representation of the changes in population of the studied cell types. Vertical red line shows the time of birth. RPKM = reads per kilobase million.

To explore how a population of neural cell types (such as neural stem or progenitor cells astrocytes, oligodendrocytes, microglia, and neurons) could be changed during developmental stages of PFC, expression trajectories of their specific markers were analyzed. Results showed that the highest expression of stem or progenitor cell markers is belonged to the early prenatal stages of PFC development (Figure 7(a)). Neuronal cell markers started to be increased at the middle stages (around the time of birth) (Figure 7(b)). Also, the markers of microglial cells showed an increase in expression level at most of the stages of PFC development (Figure 7(c)), suggesting the active immune system throughout these stages. The expression of astrocyte and oligodendrocyte markers was observed to be increased at the late stages of PFC development (Figure 7(d) and (e)). According to these data, we can conclude that development of PFC is associated with changes in population of particular cell types (Figure 7(f)).

### The Numbers or FCs of DEGs Are Not Due to Alterations in Global Transcription Rate

**Figure 8.**
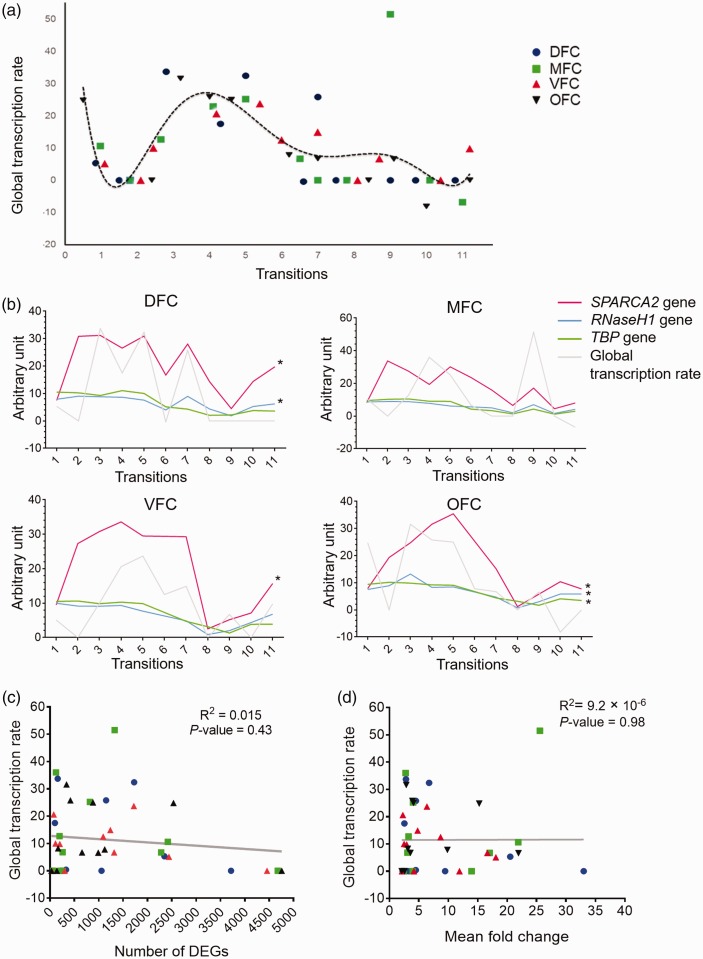
Global transcription rate across prefrontal cortex development and its correlation with numbers and FCs of DEGs. (a) Distribution of biological processes related to global transcription regulation across developmental transitions of DFC (blue), MFC (green), VFC (red), and OFC (black). The regression line represented the trajectory of global transcription rate across prefrontal cortex development. (b) Expression trajectories of three transcription factor genes across the four regions of prefrontal cortex. As shown, there is a significant correlation between the trajectories of global transcription rate and expression level alterations of the examined genes (*p* < .05). (c) Correlation analysis between the level of global transcription rate and the numbers of DEGs. (d) Correlation analysis between the level of global transcription rate and the mean fold changes of DEGs. DFC = dorsolateral prefrontal cortex; MFC = medial prefrontal cortex; VFC = ventrolateral prefrontal cortex; OFC = orbital frontal cortex.

To investigate if the number or FCs of DEGs in developing PFC is modulated by general transcriptional regulatory system, first, the biological processes which are linked to RNA polymerase (RNA-pol) activity (Table 1) were taken into account and a scatter plot was generated. Analysis of the scatter plot by nonlinear regression method showed a convergence distribution (*p* = .04) with a dynamic pattern. Also, these data demonstrated highest rates of global transcription for the Transitions 4 and 5 of PFC development (Figure 8(a)). The lowest rate of global transcription was observed for the earliest and latest stages of PFC development. However, the global transcription rate in OFC at Transition 1 and Transition 9 in MFC showed a different pattern. Moreover, the observed global transcription rate was significantly correlated with the expression trajectory of at least one of the TFs TBP, RNase-H1, and SPARCA2 (Figure 8(b)). However, due to the odd data of the global transcription rate of the MFC at Transition 9, the calculated correlation was not significant (Figure 8(b)). The detailed correlation data of these analyses are in the Supplemental File S4.

To investigate if the RNA polymerase-related biological processes (Table 1) are responsible for the architecting DEGs during development of PFC, the correlation between the numbers and FC magnitude of DEGs and global transcription rates was analyzed. Results showed no significant correlation between global transcription rates and numbers (*R*^2^ = .015, *p* = .43) or mean FCs of DEGs (*R*^2^ = 9.2 × 10^−6^, *p* = .98) (Figure 8(c) and (d), respectively). These data suggest that general transcriptional regulatory system could not be the main determinant of DEGs profile across development of PFC.

### Biological Processes That Underlie the Dynamic Transcriptome of PFC During Development

**Figure 9.**
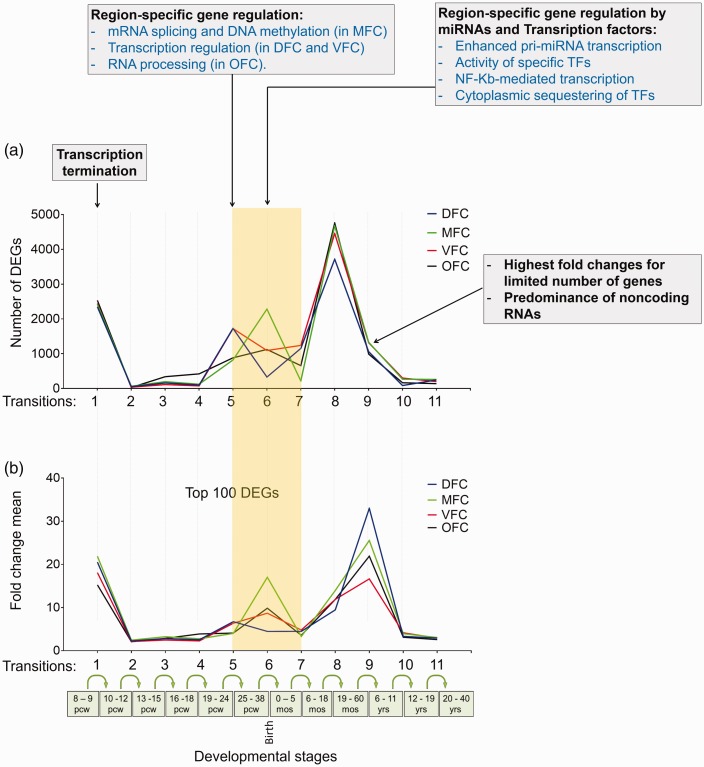
Transcription landscape of prefrontal cortex, across 11 developmental transitions and its relation to biological processes. (a) The number of DEGs that were calculated by differential expression analysis of the BrainSpan, by considering Log_2_ FC ≥ 2. (b) The mean fold changes calculated for the top 100 DEGs in each developmental stages. The yellow-shaded box denotes the perinatal stages around the Transition 6. The text boxes on the top of graphs represent the biological processes which potentially affect the observed numbers or fold change values of the DEGs. DFC = dorsolateral prefrontal cortex; MFC = medial prefrontal cortex; VFC = ventrolateral prefrontal cortex; OFC = orbital frontal cortex.

As shown before, regulatory processes of global transcription are not the underlying mechanisms for dynamic transcriptome of PFC during development (Figure 8(c) and (d)). Therefore, we decided to compare the other enriched biological processes (Supplemental File S2; *p* < .05) with numbers and mean FCs of DEGs in developmental stages of PFC. Results not only depicted dynamic feature of PFC transcriptome during development but also highlighted several biological processes that potentially may affect both frequencies (numbers) and FCs of DEGs (Figure 9). Such variations in number and FCs of DEGs during PFC development represent dynamic characteristic of PFC transcriptome.

In addition, comparison of the transcriptome landscape between four regions of PFC depicted a similar pattern with the exception of a window of meaningfully diverse transcriptome between Transitions 5 and 7 (from 25 pcw to 5 months after birth) (Figure 9; Transitions 5–7). Through analysis of the enriched biological processes in these transitions (Supplemental File S2), we found the biological process with regulatory roles on gene expression and, interestingly, they were enriched in a region-specific manner.

As shown in Figure 9, the biological process of transcription termination (at Transition 1) is occurred commonly in all prefrontal regions, thus it may alter their transcriptome, coordinately. Unlikely, between Transitions 5 and 7, transcriptome of the regions was diverse but concordant with region-specific regulatory processes such as regulation of mRNA splicing and DNA methylation in MFC, transcription regulation in DFC and VFC, and regulation of RNA processing in OFC (Figure 9; Stage 5). During Transition 6, the gene regulatory systems mediated by microRNAs and TFs were significantly enriched (*p* < .05) which may differentially alter the transcriptome of each region of PFC (Figure 9; Stage 6).

On the other hand, transcription landscape of the next transitions pointed out the Transition 9 harboring ∼1,000 DEGs with the greatest mean FC (∼20). By looking at the list of these DEGs, we found that the greatest number of them are noncoding RNAs of different classes (Supplemental File S1). A similar landscape was observed when downsampling method was applied on the raw RNA-seq data of the BrainSpan (Supplemental File S5-A), which also showed a significant correlation between the number of DEGs, calculated by these two different methods (*R*^2^ = .8684; *p* < .0001) (Supplemental File S5-B).

### Potential Signaling Pathways Involved in the Development of PFC

To find the signaling pathways potentially involved in the development of PFC, enrichment analysis of signaling pathways was performed in DAVID software using the DEGs of each developmental transition as input. Results showed a list of signaling pathways which differ in their specificity to the regions or developmental times of PFC (Table 2). Due to such time- and region-specific enrichments, a set of signaling pathways could be considered as a signature for each developmental transition or region of PFC.

**Table 2.** The Signaling Pathways Predicted to Be Involved in Development of Prefrontal Cortex Regions.

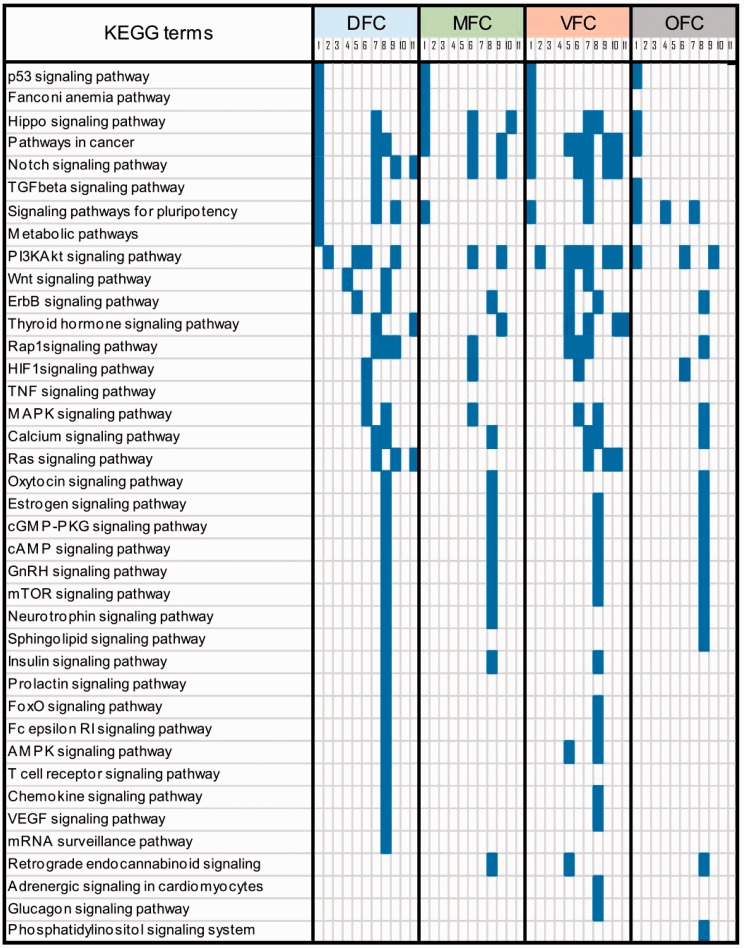

*Note.* The four studied regions of prefrontal cortex are shown as DFC, MFC, VFC, and OFC. Each region of prefrontal cortex contains 12 developmental transitions (numbered as 1–11). The filled cells denote the enrichment and the blank ones denote lack of enrichment of each pathway. As shown, signaling pathways are distributed across the table in a time-and region-dependent manner. These data show that some of the pathways are common and the other ones are specific to particular regions or developmental transitions of prefrontal cortex. For enrichment analysis, the DAVID software was used and a *p* < 0.05 was considered as statistically significant. KEGG = Kyoto Encyclopedia of Genes and Genomes; DFC = dorsolateral prefrontal cortex; MFC = medial prefrontal cortex; VFC = ventrolateral prefrontal cortex; OFC = orbital frontal cortex; VEGF = Vascular endothelial growth factor; TNF = tumor necrosis factor; PKG = Protein Kinase G; mTOR = mammalian target of rapamycin; MAPK = mitogen-activated protein kinase.

### Comparison of the Human PFC With Other Brain Regions Based on Their DEGs and Biological Processes

**Figure 10.**
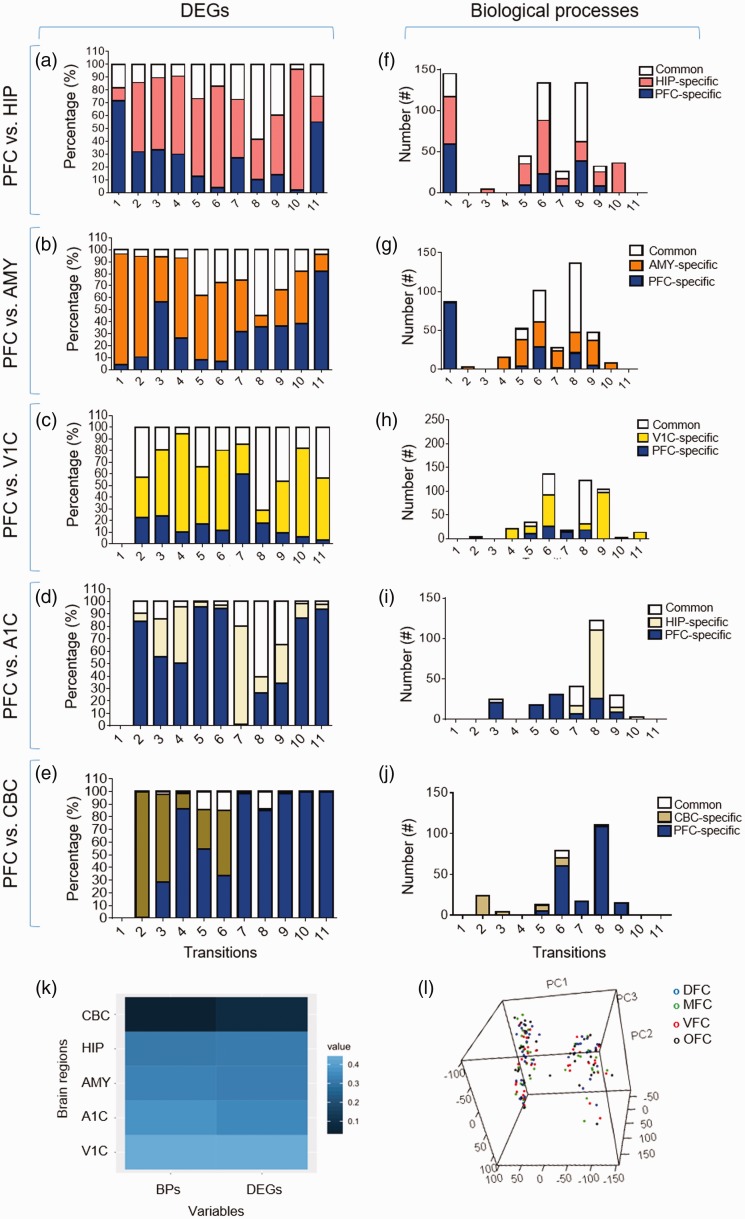
Comparison of the DEGs and BPs of prefrontal cortex with other brain regions. (a–e) Comparison between prefrontal cortex and other brain regions based on their similarity (or differences) in DEGs. (f–j) Comparison between prefrontal cortex and other brain regions based on their similarity (or differences) in biological processes. (k) A heatmap which represents the degree of differences between prefrontal cortex and other studied brain regions. As shown, CBC is the farthest and the V1C is the closest region to the prefrontal cortex based on both BPs and DEGs. (l) Three-dimensional PCA plot of gene expression data. Each color represents each region of prefrontal cortex (blue: DFC, green: MFC, red: VFC and black: OFC.). The *x*-, *y*-, and *z*-axes represent principal components 1, 2, and 3, respectively. DEG = differentially expressed genes; PFC = prefrontal cortex; HIP = hippocampus; AMY = amygdala; V1C = primary visual cortex; CBC = cerebellum cortex; BP = biological process.

To investigate if DEGs and their corresponding biological processes could discriminate PFC from other regions of human brain, Venn diagram analysis was performed. For this purpose, the lists of DEGs and biological processes of five regions including HIP, amygdala (AMY), primary visual cortex (V1C), primary auditory cortex (A1C), and cerebellum cortex (CBC) (Supplemental File S6) were compared with those of PFC. Results showed that the studied brain regions can be distinguished based on their DEGs (Figure 10(a) to (e)) and biological processes (Figure 10(f) to (j)). Also, we found that the similarity degree between these brain regions could be deduced from their lists of DEGs and BPs (Figure 10(k)). However, the PCA on the transcriptome data of PFC could not segregate DFC, MFC, VFC, or OFC regions (Figure 10(l)), suggesting their similar transcriptome.

## Discussion

Brain development is a multistage process, accompanied by coordinated expression of many genes (G.-Z. Wang and Konopka, 2013). Thanks to availability of spatiotemporal transcriptome of the human brain by BrainSpan (www.brainspan.org); in this study, we investigated to find biological processes as well as signaling pathways, correspondingly involved in the development of PFC of human brain. Thereafter, biological processes that underlie the dynamic feature of PFC transcriptome during its development were explored. Moreover, the observed developmental processes of the PFC were compared with some other brain regions to evaluate their specificity.

As an important executive part of the human brain, the PFC is the main focus of this study. PFC occupies one third of the entire human cerebral cortex and it can be divided into four sections: DFC, MFC, VFC, and OFC (Siddiqui et al., 2008) which are structurally connected while functionally distinct (Ghashghaei and Barbas, 2001; Johansen-Berg et al., 2004). Due to such characteristics and also its previously mentioned prominent role in normal brain physiology (Fuster, 2001; Miller and Cohen, 2001; Lara and Wallis, 2015) and several human psychiatric phenotypes (Fuster, 2001; Lagopoulos et al., 2012), we were interested to find molecular mechanisms underlying its development. To this end, RNA-seq data of the BrainSpan database were used to determine DEGs between subsequent developmental stages of PFC (Figure 1). For validation of the DEGs, the similar approaches were performed on microarray data. In addition, using downsampling method on the raw data of RNA-seq in BraiSpan, we confirmed the identity of the DEGs for downstream analyses.

After the identification and validation of the DEGs, gene set enrichment analysis was performed by DAVID software and the R package WGCNA in order to derive biological processes and signaling pathways potentially involved in development of PFC. Through interpretation of the results of DAVID and WGCNA, the developmental transitions of PFC with their enriched biological processes are demonstrated in Figures 2(c) to 5(c). As shown in Table 3, the first developmental transition (around 10th pcw) of PFC was predicted to be mediated by the biological processes that are restricted to DNA metabolism and cell division (or mitosis) which are characteristic features of neural stem or progenitor cells (Murao et al., 2016).

**Table 3.** The Biological Processes Which Were Commonly Enriched for All Prefrontal Cortex Regions.

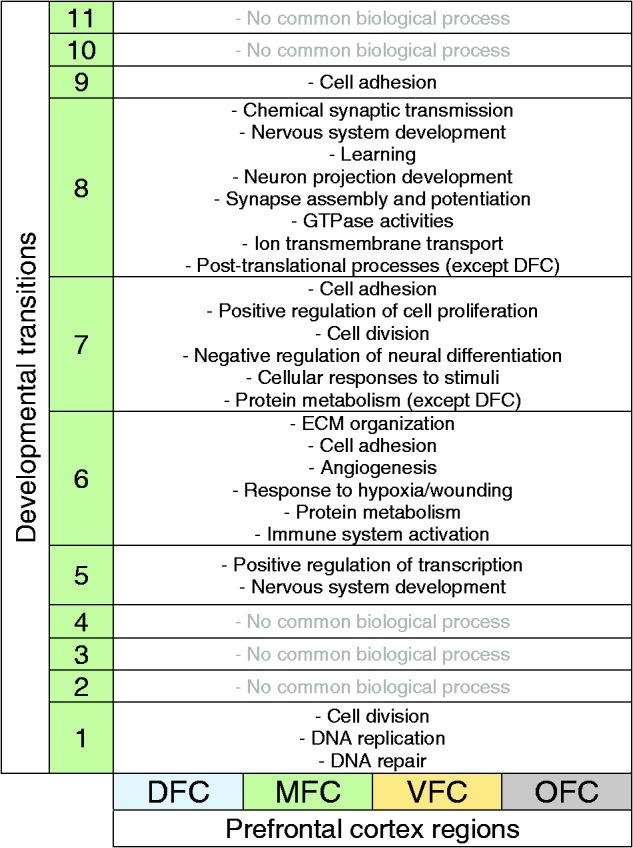

*Note.* These biological processes are not region-specific and may orchestrate the development of prefrontal cortex regions. Each number in the column 2 denotes the developmental transitions of 1-11. DFC= dorsolateral prefrontal cortex; MFC= medial prefrontal cortex; VFC= ventrolateral prefrontal cortex; OFC= orbital frontal cortex; ECM= extracellular matrix.

The second series of commonly enriched biological processes were “positive regulation of transcription” and “nervous system development,” occurring at the developmental Transition 5 (around 24th pcw) (Table 3). Notably, the biological processes of this transition are closely similar between all PFC regions.

Transition from Stages 6 to 7 (time of birth) is concurrent with “ECM organization,” “cell adhesion,” “angiogenesis,” “response to hypoxia or wounding,” and “protein metabolism” for all regions. Nevertheless, the difference is that the “translation initiation” (as a part of protein metabolism) is started in MFC, VFC, and OFC, but not still started in DFC (Table 3). Also, biological processes related to immune system activation (“platelet degranulation” and “leukocyte migration”) are also common for all PFC, implying that immune system is particularly important during Transition 6 (i.e., around the time of birth: 38 pcw). Consistently, increasing evidence have shown the causative role of perinatal immune system malfunctioning in neuropsychiatric conditions (Hagberg and Mallard, 2005; Missig et al., 2018). Expression analysis of the specific marker of microglial cells across developmental transition of PFC region emphasizes on the remarkable increased level of these cells (and immune system activity) at the time of birth (Transition 6) (Figure 7(c)).

In comparison with other developmental transitions, the biological processes of Transition 8 (between the Stages 8 and 9) are more similar throughout DFC, MFC, VFC, and OFC. In fact, transition from Stages 8 to 9 mostly requires some processes such as “chemical synaptic transmission,” “nervous system development,” “learning,” “neuron projection development,” “synapse assembly and potentiation,” “GTPase activities,” and “ion transmembrane transport”. However, regarding the ion transportation, the only enriched biological process for DFC was “calcium ion transport.” In addition, GTPase activities can drive many signaling pathways within neurons (Aspenström, 2004; Stankiewicz and Linseman, 2014); therefore, it is acceptable that the greatest number of signaling pathways are particularly enriched for the DEGs of the Transition 8 (see Table 2). Moreover, unlike in previous transitions where protein metabolism was restricted to rRNA processing, NMD and translation initiation, during this time, posttranslational processes such as “protein ubiquitination,” “proteasome-mediated catabolic process,” and “intracellular protein transport” were enriched. Such posttranslational processes, however, were not still enriched for DFC that again confirmed the delay in protein metabolism of this region (Table 3; green highlighted). Consistently, in a previous study, comparative proteome analysis of PFC of nine schizophrenia and seven healthy individuals showed an altered protein profile in DFC among all PFC regions (Martins-de-Souza et al., 2009).

In the Year 5 of human brain development (Transitions 9–11), the biological processes vary greatly between PFC regions so that “cell adhesion” is the only common biological process of Transition 9 shared between all regions while the other enriched biological processes are region specific.

By comparing the mean FC of DEGs throughout all developmental transitions, we found that the developmental Transition 9 of all regions possessed the highest FC value (Figure 9). Analysis of the DEGs in this transition revealed that most of the DEGs are noncoding RNAs (ncRNAs). In fact, these DEGs encode vast variety of ncRNA classes such as ribosomal RNAs (rRNAs), pseudogene RNAs, long noncoding RNAs (lncRNAs), small nuclear RNAs (snRNAs), small nucleolar RNAs (snoRNAs), small cytoplasmic RNAs (scRNAs), and so forth (Supplemental File S1). The evident regulatory role of ncRNAs (Wery et al., 2011) and their differential expression during Transition 9 (∼5 years after birth) highlights their necessity in functionality of developed PFC. A comprehensive study by He et al. (2014) showed low but ever-changing expression of lncRNAs across developmental time series of human PFC. Consistent with our results, they also found that a significant expression alteration of lncRNAs predominantly occurs in late developmental stages (∼9 years after birth) of PFC (He et al., 2014). The essential role of such regulatory RNAs is reflected in almost every aspect of neural system behavior, including chromatin modification (by Y RNAs) (Kowalski and Krude, 2015), transcriptional regulation (by lncRNAs) (Long et al., 2017), alternative splicing (by snRNAs) (Karijolich and Yu, 2010), RNA editing (by snoRNAs) (Vitali et al., 2005), and translation (by rRNAs, Dahlberg, 1989, and miRNAs, Fabian et al., 2010).

Another support for our result is provided by the correlation between the increases of the noncoding transcriptome and cognitive evolution in primates (Guennewig and Cooper, 2014). Such abundance of ncRNAs in developed PFC supports the pivotal role of ncRNAs in adult brain functioning. However, experimental studies are needed to determine if these changes in transcription of ncRNAs are cause or subsequence of PFC development.

In addition to pivotal role of common biological processes in PFC (Table 3), some biological processes were enriched in a region-specific manner (Table 4). Such processes can be considered as a molecular signature for each region and may have a central role for specification of the regions.

**Table 4.** Comparison of the Biological Processes Involved in the Development of Distinct Regions of Human Prefrontal Cortex.

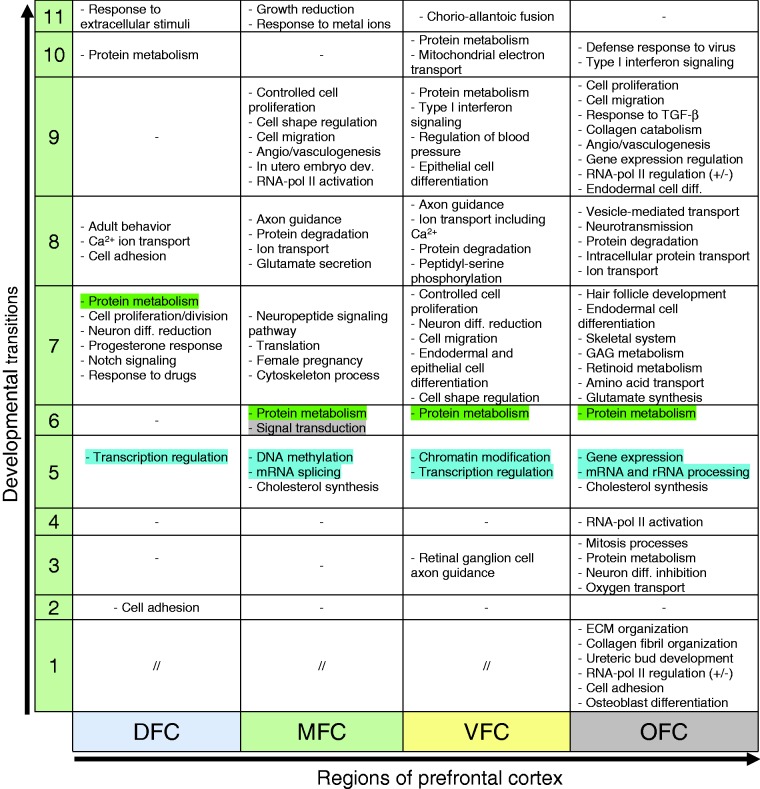

*Note.* The “−” and “//”denotes no enrichment of biological process and the commonly enriched biological processes, respectively. Each number in the column 1 denotes the developmental transitions of 1–11. DFC = dorsolateral prefrontal cortex; MFC = medial prefrontal cortex; VFC = ventrolateral prefrontal cortex; OFC= orbital frontal cortex; GAG = glycosaminoglycan; ECM = extracellular matrix.

As shown in Table 4, among all regions, the OFC showed unique set of biological processes across initial transitions (1–4) of PFC development: “Osteoblast differentiation,” “cell adhesion,” “RNA-pol II regulation,” “Ureteric acid development,” “collagen fibril organization,” and “ECM organization” (in Transition 1); mitosis processes, protein metabolism, neuron differentiation inhibition, and oxygen transport (in Transition 3); and RNA-pol II activation (in Transition 4) (Table 4). Studies showed that among all regions of PFC, the OFC receives environmental stimuli or experiences in early developmental stages and accordingly formulates behavioral characteristics (Bachevalier and Loveland, 2006; Hanson et al., 2010). Therefore, this unique set of biological processes which were enriched for OFC may mediate such events.

In early stages of VFC development, the biological process of “axon guidance of retinal ganglion cells” was specifically enriched (Table 4) which is consistent with the positional proximity of VFC to the human retina (Romanski, 2007).

In developmental Transition 5 of PFC, the region-specific biological processes are mainly linked to gene expression regulation, whose regulatory effects differ between regions (Table 4; blue highlighted). For example, enrichment of DNA methylation” and “mRNA splicing via spliceosome” for MFC, “covalent chromatin modification” for VFC, and “regulation of transcription from RNA-pol II” for OFC. Such varied regulatory systems could be the causes of observed diverse transcription profile of each region in this developmental transition (Figure 9). In addition, “cholesterol biosynthetic process” is a cellular event of the Transition 5, which was enriched specifically for MFC and OFC (Table 4). Studies also proved the relationship between cholesterol metabolism and central nervous system development (L. Wang et al., 2002; Xu et al., 2014; Courtney and Landreth, 2016).

During Transition 6 (the time of birth), while most biological processes are common in all PFC regions, the biological processes of protein metabolism (“rRNA processing,” “translational initiation,” “NMD,” and “signal recognition particle [SRP]-mediated protein targeting to membrane”) were enriched for MFC, VFC, and OFC but not DFC. In DFC, these processes emerge in Transition 7, suggesting existence of different protein profile in each region of PFC.

In Transition 7, the common hallmark of all regions is balanced between cell proliferation and neural differentiation. However, type and rate of cell proliferation and differentiation are different across regions. Similarly, in Transition 7, the biological process of cellular response to several stimuli is a common characteristic of all regions but depending on the region, stimulus may be a hormone, drug, wounding, or hypoxia (Table 4). According to these enriched biological processes for Transition 7, a prominent event could be ‘neurogenesis’ which requires both cell proliferation (of neural stem or progenitor cells) and differentiation under a tightly regulated process (Kirschenbaum et al., 1994). While it is found that neurogenesis mostly occurs in HIP, studies showed that PFC also harbors progenitor cells in which the neurogenesis can be induced by the aforementioned stimuli (Fuchs, 2008). Expression analysis of the neuronal cell markers also confirmed the active process of neurogenesis during Transitions 5 to 11 (Figure 7(b)). In addition, among all PFC regions, MFC and OFC were previously illustrated to be more influenced by environmental stimuli. However, nearly always, their responses are in opposite directions (Kolb et al., 2012).

The Transition 8 contains many biological processes, commonly enriched for all regions of PFC (Table 3). Nevertheless, the region-specific biological processes were mostly related to nervous system activity and behavior. Interestingly, protein synthesis and metabolism which were emerged and continued through the Transitions 6 and 7 become declined in the Transition 8 of MFC, VFC, and OFC (but not DFC) via processes of protein degradation. As mentioned, translational initiation in DFC begins later than that of other regions; expectedly, protein degradation is not enriched within the DFC yet (Table 4).

Transitions 9 to 11 of PFC (from 5 to 40 years after birth) harbor the most differential biological processes: In DFC, cell adhesion process is followed by protein metabolism and cellular response to extracellular stimulus; in MFC, the biological processes related to angiogenesis or vasculogenesis, ECM organization, cell adhesion, cell proliferation, maturation, migration, and shaping (in Transition 9) are followed by negative regulation of growth and cellular response to zinc, cadmium, and other metallic ions (in Transition 11). In VFC, other than the role of protein metabolism processes in Transitions 9 and 10, three physiologically important processes (i.e., Type-I interferon signaling pathway, regulation of blood pressure, and mitochondrial electron transport) are emerged. In OFC, ECM organization, cell adhesion, proliferation, migration and differentiation, angiogenesis or vasculogenesis were observed, which also were in common with MFC. However, cellular response to TGF-β stimulus, collagen catabolic process, gene expression regulation (in Transition 9) together with “defense response to virus” and “Type-I interferon signaling pathway” (in Transition 10) were unique.

In addition to comparison of two immediate developmental stages to acquire DEGs, the lists of coexpressed genes during PFC development was obtained by WGCNA and DAVID software predicted their corresponding biological processes. Many of the resultant biological processes were similar to the results of DEG analysis, while other processes were different (Figure 6).

We were interested to evaluate the transcriptome landscape of PFC during development. To this purpose, biological processes related to RNA polymerase activity were considered and scored based on their percentage (%) in the results of enrichment analyses (Supplemental File S2 and Table 1). Analyses showed that in spite of some deviations, global transcription rates of PFC regions during development have a convergent but dynamic pattern (*p* < .05) (Figure 8(a)). The dynamic feature of PFC transcriptome was also verified by the expression trajectories of three general TFs (Figure 8(b)). Next, we investigated if the global transcription rate affects number or FC values of DEGs. The results showed no significant correlation between global transcription rate and number or FCs of DEGs (Figure 8(c) and (d)), suggesting that regulatory systems of global transcription (e.g., RNA polymerases and their regulators) are not the effectors to change PFC transcriptome. For this reason, we tried to find additional biological processes that underlie dynamic feature of PFC transcriptome during development. Interestingly, according to the enriched biological processes (Supplementary File S2), a concordance between transcriptome landscape and several biological processes was found (Figure 9). In detail, the biological processes of “transcription termination” are simultaneous with the decrease in number and FCs of DEGs in Transition 1 (Figure 9). The Transitions 5, 6, and 7 exhibited diverse transcriptome which are concurrent with regulatory mechanisms which were particularly enriched in a region-specific manner. Such differential regulatory systems confer a remarkable heterogeneity to transcriptomes of the PFC regions in Transition 5 (Figure 9). During Transition 6, a more specific regulatory system is launched by miRNAs and TFs. Although the terminology of such biological processes is common, but each region may harbor different miRNAs or TFs which subsequently can confer a different transcriptome to each region. Consistently, recent studies revealed distinct profiles of miRNAs (Ziats and Rennert, 2014) and TFs (Pfaffenseller et al., 2016) across developmental times and regions of human PFC. Like early stages of PFC development (Transitions 1 to 4), latest stages of PFC development (Transitions 8 to 11) had similar transcriptome dynamics (Figure 9).

In addition to the biological processes, several signaling pathways were also identified for development of PFC (Table 2). Such profile of signaling pathways is important specifically to speculate about the outcome(s) of deregulations in any signaling pathway when human brain is developing (Kloet and Derijk, 2004; Noelanders and Vleminckx, 2017). Moreover, the profile of signaling pathways may be helpful to design a targeted system (e.g., drug, siRNA, etc.) against the deregulated pathways to treat brain malformations (Lewandowski et al., 2016). An interesting instance is the causative role of defective insulin signaling pathway in several brain disorder (Schubert et al., 2003; Bedse et al., 2015) which could be corrected by insulin treatment (Jolivalt et al., 2008; Folch et al., 2018). While we showed that the studied PFC regions differ in their developmental processes and signaling pathways, PCA results revealed that the transcriptome profiles of these regions are not distinct (Figure 10(l)). To investigate the amount of similarities between PFC and other brain regions in the view of their molecular mechanisms during development, the lists of the DEGs for several brain regions were obtained from BrainSpan and were analyzed by DAVID. Venn diagram analysis demonstrated a divergence profiles between PFC and V1C, A1C, AMY, HIP, and CBC (Figure 10(k)). The degree of these divergences is completely consistent with the results of another study which compared the transcriptome of most human brain regions by PCA (Willsey et al., 2013). In this study we exploited the available data sets of human PFC transcriptome to identify its molecular event during development. It is of great importance to perform a similar procedure for other parts of human brain.

## Supplemental Material

Supplemental Material1 - Supplemental material for Identification of the Molecular Events Involved in the Development of Prefrontal Cortex Through the Analysis of RNA-Seq Data From BrainSpan Click here for additional data file. Supplemental material, Supplemental Material1 for Identification of the Molecular Events Involved in the Development of Prefrontal Cortex Through the Analysis of RNA-Seq Data From BrainSpan by Hadi Najafi, Mohadeseh Naseri, Javad Zahiri, Mehdi Totonchi and Majid Sadeghizadeh in ASN Neuro

## Supplemental Material

Supplemental Material2 - Supplemental material for Identification of the Molecular Events Involved in the Development of Prefrontal Cortex Through the Analysis of RNA-Seq Data From BrainSpan Click here for additional data file. Supplemental material, Supplemental Material2 for Identification of the Molecular Events Involved in the Development of Prefrontal Cortex Through the Analysis of RNA-Seq Data From BrainSpan by Hadi Najafi, Mohadeseh Naseri, Javad Zahiri, Mehdi Totonchi and Majid Sadeghizadeh in ASN Neuro

## Supplemental Material

Supplemental Material3 - Supplemental material for Identification of the Molecular Events Involved in the Development of Prefrontal Cortex Through the Analysis of RNA-Seq Data From BrainSpan Click here for additional data file. Supplemental material, Supplemental Material3 for Identification of the Molecular Events Involved in the Development of Prefrontal Cortex Through the Analysis of RNA-Seq Data From BrainSpan by Hadi Najafi, Mohadeseh Naseri, Javad Zahiri, Mehdi Totonchi and Majid Sadeghizadeh in ASN Neuro

## Supplemental Material

Supplemental Material4 - Supplemental material for Identification of the Molecular Events Involved in the Development of Prefrontal Cortex Through the Analysis of RNA-Seq Data From BrainSpan Click here for additional data file. Supplemental material, Supplemental Material4 for Identification of the Molecular Events Involved in the Development of Prefrontal Cortex Through the Analysis of RNA-Seq Data From BrainSpan by Hadi Najafi, Mohadeseh Naseri, Javad Zahiri, Mehdi Totonchi and Majid Sadeghizadeh in ASN Neuro

## Supplemental Material

Supplemental Material5 - Supplemental material for Identification of the Molecular Events Involved in the Development of Prefrontal Cortex Through the Analysis of RNA-Seq Data From BrainSpan Click here for additional data file. Supplemental material, Supplemental Material5 for Identification of the Molecular Events Involved in the Development of Prefrontal Cortex Through the Analysis of RNA-Seq Data From BrainSpan by Hadi Najafi, Mohadeseh Naseri, Javad Zahiri, Mehdi Totonchi and Majid Sadeghizadeh in ASN Neuro

## Supplemental Material

Supplemental Material6 - Supplemental material for Identification of the Molecular Events Involved in the Development of Prefrontal Cortex Through the Analysis of RNA-Seq Data From BrainSpan Click here for additional data file. Supplemental material, Supplemental Material6 for Identification of the Molecular Events Involved in the Development of Prefrontal Cortex Through the Analysis of RNA-Seq Data From BrainSpan by Hadi Najafi, Mohadeseh Naseri, Javad Zahiri, Mehdi Totonchi and Majid Sadeghizadeh in ASN Neuro

## References

[bibr1-1759091419854627] Abé C. Ekman C.-J. Sellgren C. Petrovic P. Ingvar M. Landén M. (2015). Manic episodes are related to changes in frontal cortex: A longitudinal neuroimaging study of bipolar disorder 1. Brain, 138(11), 3440–3448. 26373602 10.1093/brain/awv266

[bibr2-1759091419854627] Anderson M. L. Kinnison J. Pessoa L. (2013). Describing functional diversity of brain regions and brain networks. Neuroimage, 73, 50–58. 23396162 10.1016/j.neuroimage.2013.01.071 PMC3756684

[bibr3-1759091419854627] Artegiani B. Lyubimova A. Muraro M. van Es J. H. van Oudenaarden A. Clevers H. (2017). A single-cell RNA sequencing study reveals cellular and molecular dynamics of the hippocampal neurogenic niche. Cell Rep, 21(11), 3271–3284. 29241552 10.1016/j.celrep.2017.11.050

[bibr4-1759091419854627] Aspenström P. (2004). Integration of signalling pathways regulated by small GTPases and calcium. Biochimica Biophys Acta (BBA)-Molecular Cell Research, 1742(1–3), 51–58. 10.1016/j.bbamcr.2004.09.029 15590055

[bibr5-1759091419854627] Bachevalier J. Loveland K. A. (2006). The orbitofrontal–Amygdala circuit and self-regulation of social–emotional behavior in autism. Neurosci Biobehav Rev, 30(1), 97–117. 16157377 10.1016/j.neubiorev.2005.07.002

[bibr6-1759091419854627] Bedse G. Di Domenico F. Serviddio G. Cassano T. (2015). Aberrant insulin signaling in Alzheimer's disease: Current knowledge. Front Neurosci, 9, 204. 26136647 10.3389/fnins.2015.00204 PMC4468388

[bibr7-1759091419854627] Cahoy J. D. Emery B. Kaushal A. Foo L. C. Zamanian J. L. Christopherson K. S. Xing Y. Lubischer J. L. Krieg P. A. Krupenko S. A. (2008). A transcriptome database for astrocytes, neurons, and oligodendrocytes: A new resource for understanding brain development and function. J Neurosci, 28(1), 264–278. 18171944 10.1523/JNEUROSCI.4178-07.2008 PMC6671143

[bibr8-1759091419854627] Clark L. Sahakian B. J. (2008). Cognitive neuroscience and brain imaging in bipolar disorder. Dialogues Clin Neurosci, 10(2), 153. 18689286 10.31887/DCNS.2008.10.2/lclark PMC3181872

[bibr9-1759091419854627] Courtney R. Landreth G. E. (2016). LXR regulation of brain cholesterol: From development to disease. Trends Endocrinol Metab, 27(6), 404–414. 27113081 10.1016/j.tem.2016.03.018 PMC4986614

[bibr10-1759091419854627] Dahlberg A. E. (1989). The functional role of ribosomal RNA in protein synthesis. Cell, 57(4), 525–529. 2655923 10.1016/0092-8674(89)90122-0

[bibr11-1759091419854627] Erraji-Benchekroun L. Underwood M. D. Arango V. Galfalvy H. Pavlidis P. Smyrniotopoulos P. Mann J. J. Sibille E. (2005). Molecular aging in human prefrontal cortex is selective and continuous throughout adult life. Biol Psychiatry, 57(5), 549–558. 15737671 10.1016/j.biopsych.2004.10.034

[bibr12-1759091419854627] Fabian M. R. Sonenberg N. Filipowicz W. (2010). Regulation of mRNA translation and stability by microRNAs. Annu Rev Biochem, 79, 351–379. 20533884 10.1146/annurev-biochem-060308-103103

[bibr13-1759091419854627] Folch J. Ettcheto M. Busquets O. Sánchez-López E. Castro-Torres R. D. Verdaguer E. Manzine P. R. Poor S. R. García M. L. Olloquequi J. (2018). The implication of the brain insulin receptor in late onset Alzheimer’s disease dementia. Pharmaceuticals, 11(1), 11. 10.3390/ph11010011 PMC5874707 29382127

[bibr14-1759091419854627] Frith C. Dolan R. (1996). The role of the prefrontal cortex in higher cognitive functions. Brain Res Cogn Brain Res, 5(1–2), 175–181. 9049084 10.1016/s0926-6410(96)00054-7

[bibr15-1759091419854627] Fuchs E. (2008, August). *Neurogenesis in the adult brain: The association with stress in depression*. 21st Congress of the European College of Neuropsychopharmacology, Barcelona, Spain.

[bibr16-1759091419854627] Fuster J. M. (2001). The prefrontal cortex—An update: Time is of the essence. Neuron, 30(2), 319–333. 11394996 10.1016/s0896-6273(01)00285-9

[bibr17-1759091419854627] Ghashghaei H. Barbas H. (2001). Neural interaction between the basal forebrain and functionally distinct prefrontal cortices in the rhesus monkey. Neuroscience, 103(3), 593–614. 11274781 10.1016/s0306-4522(00)00585-6

[bibr18-1759091419854627] Goldberg M. C. Spinelli S. Joel S. Pekar J. J. Denckla M. B. Mostofsky S. H. (2011). Children with high functioning autism show increased prefrontal and temporal cortex activity during error monitoring. Dev Cogn Neurosci, 1(1), 47–56. 21151713 10.1016/j.dcn.2010.07.002 PMC2999812

[bibr19-1759091419854627] Goldstein R. Z. Volkow N. D. (2011). Dysfunction of the prefrontal cortex in addiction: Neuroimaging findings and clinical implications. Nat Rev Neurosci, 12(11), 652. 22011681 10.1038/nrn3119 PMC3462342

[bibr20-1759091419854627] Guennewig B. Cooper A. A. (2014). The central role of noncoding RNA in the brain. Int Rev Neurobiol, 116, 153–194. 25172475 10.1016/B978-0-12-801105-8.00007-2

[bibr21-1759091419854627] Hagberg H. Mallard C. (2005). Effect of inflammation on central nervous system development and vulnerability. Curr Opin Neurol, 18(2), 117–123. 15791140 10.1097/01.wco.0000162851.44897.8f

[bibr22-1759091419854627] Hanson J. L. Chung M. K. Avants B. B. Shirtcliff E. A. Gee J. C. Davidson R. J. Pollak S. D. (2010). Early stress is associated with alterations in the orbitofrontal cortex: A tensor-based morphometry investigation of brain structure and behavioral risk. J Neurosci, 30(22), 7466–7472. 20519521 10.1523/JNEUROSCI.0859-10.2010 PMC2893146

[bibr23-1759091419854627] He Z. Bammann H. Han D. Xie G. Khaitovich P. (2014). Conserved expression of lincRNA during human and macaque prefrontal cortex development and maturation. RNA, 20(7), 1103–1111. 24847104 10.1261/rna.043075.113 PMC4074677

[bibr24-1759091419854627] Johansen-Berg H. Behrens T. Robson M. Drobnjak I. Rushworth M. Brady J. Smith S. Higham D. Matthews P. (2004). Changes in connectivity profiles define functionally distinct regions in human medial frontal cortex. Proc Natl Acad Sci U S A, 101(36), 13335–13340. 15340158 10.1073/pnas.0403743101 PMC516567

[bibr25-1759091419854627] Jolivalt C. Lee C. Beiswenger K. Smith J. Orlov M. Torrance M. Masliah E. (2008). Defective insulin signaling pathway and increased GSK-3 activity in the brain of diabetic mice: Parallels with Alzheimer’s disease and correction by insulin. J Neurosci Res, 86(15), 3265. 18627032 10.1002/jnr.21787 PMC4937800

[bibr26-1759091419854627] Kang H. J. Kawasawa Y. I. Cheng F. Zhu Y. Xu X. Li M. Sousa A. M. Pletikos M. Meyer K. A. Sedmak G. (2011). Spatio-temporal transcriptome of the human brain. Nature, 478(7370), 483. 22031440 10.1038/nature10523 PMC3566780

[bibr27-1759091419854627] Karijolich J. Yu Y.-T. (2010). Spliceosomal snRNA modifications and their function. RNA Biol, 7(2), 192–204. 20215871 10.4161/rna.7.2.11207 PMC4154345

[bibr28-1759091419854627] Kirschenbaum B. Nedergaardm M. Preuss A. Barami K. Fraser R. A. Goldman S. A. (1994). In vitro neuronal production and differentiation by precursor cells derived from the adult human forebrain. Cereb Cortex, 4(6), 576–589. 7703685 10.1093/cercor/4.6.576

[bibr29-1759091419854627] Kloet E. R. Derijk R. (2004). Signaling pathways in brain involved in predisposition and pathogenesis of stress‐related disease: Genetic and kinetic factors affecting the MR/GR balance. Ann N Y Acad Sci, 1032(1), 14–34. 15677393 10.1196/annals.1314.003

[bibr30-1759091419854627] Koenigs M. Grafman J. (2009). The functional neuroanatomy of depression: Distinct roles for ventromedial and dorsolateral prefrontal cortex. Behav Brain Res, 201(2), 239–243. 19428640 10.1016/j.bbr.2009.03.004 PMC2680780

[bibr31-1759091419854627] Kolb B. Mychasiuk R. Muhammad A. Li Y. Frost D. O. Gibb R. (2012). Experience and the developing prefrontal cortex. Proc Natl Acad Sci, 109(Supplement 2), 17186–17193. 23045653 10.1073/pnas.1121251109 PMC3477383

[bibr32-1759091419854627] Kotsantis P. Silva L. M. Irmscher S. Jones R. M. Folkes L. Gromak N. Petermann E. (2016). Increased global transcription activity as a mechanism of replication stress in cancer. Nat Commun, 7, 13087. 27725641 10.1038/ncomms13087 PMC5062618

[bibr33-1759091419854627] Kowalski M. P. Krude T. (2015). Functional roles of non-coding Y RNAs. Int J Biochem Cell Biol, 66, 20–29. 26159929 10.1016/j.biocel.2015.07.003 PMC4726728

[bibr34-1759091419854627] Lagopoulos J. Hermens D. F. Naismith S. L. Scott E. M. Hickie I. B. (2012). Frontal lobe changes occur early in the course of affective disorders in young people. BMC Psych, 12(1), 4. 10.1186/1471-244X-12-4 PMC3280164 22264318

[bibr35-1759091419854627] Langfelder P. Horvath S. (2008). WGCNA: An R package for weighted correlation network analysis. BMC Bioinf, 9(1), 559. 10.1186/1471-2105-9-559 PMC2631488 19114008

[bibr36-1759091419854627] Lara A. H. Wallis J. D. (2015). The role of prefrontal cortex in working memory: A mini review. Front Syst Neurosci, 9, 173. 26733825 10.3389/fnsys.2015.00173 PMC4683174

[bibr37-1759091419854627] Lewandowski S. A. Fredriksson L. Lawrence D. A. Eriksson U. (2016). Pharmacological targeting of the PDGF-CC signaling pathway for blood–brain barrier restoration in neurological disorders. Pharmacol Ther, 167, 108–119. 27524729 10.1016/j.pharmthera.2016.07.016 PMC5341142

[bibr38-1759091419854627] Long Y. Wang X. Youmans D. T. Cech T. R. (2017). How do lncRNAs regulate transcription? Sci Adv, 3(9), eaao2110. 28959731 10.1126/sciadv.aao2110 PMC5617379

[bibr39-1759091419854627] Martins-de-Souza D. Gattaz W. F. Schmitt A. Maccarrone G. Hunyadi-Gulyás E. Eberlin M. N. Souza G. H. Marangoni S. Novello J. C. Turck C. W. (2009). Proteomic analysis of dorsolateral prefrontal cortex indicates the involvement of cytoskeleton, oligodendrocyte, energy metabolism and new potential markers in schizophrenia. J Psychiatr Res, 43(11), 978–986. 19110265 10.1016/j.jpsychires.2008.11.006

[bibr40-1759091419854627] McKenzie A. T. Wang M. Hauberg M. E. Fullard J. F. Kozlenkov A. Keenan A. Hurd Y. L. Dracheva S. Casaccia P. Roussos P. (2018). Brain cell type specific gene expression and co-expression network architectures. Sci Rep, 8, 8868. 29892006 10.1038/s41598-018-27293-5 PMC5995803

[bibr41-1759091419854627] Miller E. K. Cohen J. D. (2001). An integrative theory of prefrontal cortex function. Annu Rev Neurosci, 24(1), 167–202. 11283309 10.1146/annurev.neuro.24.1.167

[bibr42-1759091419854627] Missig G. Mokler E. L. Robbins J. O. Alexander A. J. McDougle C. J. Carlezon W. A. Jr.(2018). Perinatal immune activation produces persistent sleep alterations and epileptiform activity in male mice. Neuropsychopharmacology, 43(3), 482. 28984294 10.1038/npp.2017.243 PMC5770773

[bibr43-1759091419854627] Murao N. Noguchi H. Nakashima K. (2016). Epigenetic regulation of neural stem cell property from embryo to adult. Neuroepigenetics, 5, 1–10.

[bibr44-1759091419854627] Noelanders R. Vleminckx K. (2017). How Wnt signaling builds the brain: Bridging development and disease. Neuroscientist, 23(3), 314–329. 27624848 10.1177/1073858416667270

[bibr45-1759091419854627] Pessoa L. (2014). Understanding brain networks and brain organization. Phys Life Rev, 11(3), 400–435. 24819881 10.1016/j.plrev.2014.03.005 PMC4157099

[bibr46-1759091419854627] Pfaffenseller B. da Silva Magalhães P. De Bastiani M. Castro M. A. A. Gallitano A. L. Kapczinski F. Klamt F. (2016). Differential expression of transcriptional regulatory units in the prefrontal cortex of patients with bipolar disorder: Potential role of early growth response gene 3. Transl Psychiatry, 6(5), e805. 27163206 10.1038/tp.2016.78 PMC5070056

[bibr47-1759091419854627] Romanski L. M. (2007). Representation and integration of auditory and visual stimuli in the primate ventral lateral prefrontal cortex. Cereb Cortex, 17(suppl_1), i61–i69. 17634387 10.1093/cercor/bhm099 PMC2778283

[bibr48-1759091419854627] Schubert M. Brazil D. P. Burks D. J. Kushner J. A. Ye J. Flint C. L. Farhang-Fallah J. Dikkes P. Warot X. M. Rio C. (2003). Insulin receptor substrate-2 deficiency impairs brain growth and promotes tau phosphorylation. J Neurosci, 23(18), 7084–7092. 12904469 10.1523/JNEUROSCI.23-18-07084.2003 PMC6740672

[bibr49-1759091419854627] Siddiqui S. V. Chatterjee U. Kumar D. Siddiqui A. Goyal N. (2008). Neuropsychology of prefrontal cortex. Indian J Psychiatry, 50(3), 202. 19742233 10.4103/0019-5545.43634 PMC2738354

[bibr50-1759091419854627] Silbereis J. C. Pochareddy S. Zhu Y. Li M. Sestan N. (2016). The cellular and molecular landscapes of the developing human central nervous system. Neuron, 89(2), 248–268. 26796689 10.1016/j.neuron.2015.12.008 PMC4959909

[bibr51-1759091419854627] Smyth G. K. Ritchie M. Thorne N. Wettenhall J. (2005). LIMMA: Linear models for microarray data In Bioinformatics and computational biology solutions using R and bioconductor. Statistics for biology and health (pp. 397–420). Berlin, Germany: Springer.

[bibr52-1759091419854627] Stankiewicz T. R. Linseman D. A. (2014). Rho family GTPases: Key players in neuronal development, neuronal survival, and neurodegeneration. Front Cell Neurosci, 8, 314.e 10.3389/fncel.2014.00314 PMC4187614 25339865

[bibr53-1759091419854627] Stiles J. Jernigan T. L. (2010). The basics of brain development. Neuropsychol Rev, 20(4), 327–348. 21042938 10.1007/s11065-010-9148-4 PMC2989000

[bibr54-1759091419854627] Vaidya C. J. (2011). Neurodevelopmental abnormalities in ADHD. Behavioral neuroscience of attention deficit hyperactivity disorder and its treatment (pp. 49–66). Berlin, Germany: Springer.

[bibr55-1759091419854627] Vitali P. Basyuk E. Le Meur E. Bertrand E. Muscatelli F. Cavaillé J. Huttenhofer A. (2005). ADAR2-mediated editing of RNA substrates in the nucleolus is inhibited by C/D small nucleolar RNAs. J Cell Biol, 169(5), 745–753. 15939761 10.1083/jcb.200411129 PMC2171610

[bibr56-1759091419854627] Wang G.-Z. Konopka G. (2013). Decoding human gene expression signatures in the brain. Transcription, 4(3), 102–108. 23665540 10.4161/trns.24885 PMC4042582

[bibr57-1759091419854627] Wang L. Schuster G. U. Hultenby K. Zhang Q. Andersson S. Gustafsson J.-Å. (2002). Liver X receptors in the central nervous system: From lipid homeostasis to neuronal degeneration. Proc Natl Acad Sci, 99(21), 13878–13883. 12368482 10.1073/pnas.172510899 PMC129791

[bibr58-1759091419854627] Wery M. Kwapisz M. Morillon A. (2011). Noncoding RNAs in gene regulation. Wiley Interdiscip Rev Syst Biol Med, 3(6), 728–738. 21381218 10.1002/wsbm.148

[bibr59-1759091419854627] Wible C. G. Anderson J. Shenton M. E. Kricun A. Hirayasu Y. Tanaka S. Levitt J. J. O'Donnell B. F. Kikinis R. Jolesz F. A. (2001). Prefrontal cortex, negative symptoms, and schizophrenia: An MRI study. Psychiatry Res Neuroimaging, 108(2), 65–78. 10.1016/s0925-4927(01)00109-3 PMC2845854 11738541

[bibr60-1759091419854627] Willsey A. J. Sanders S. J. Li M. Dong S. Tebbenkamp A. T. Muhle R. A. Reilly S. K. Lin L. Fertuzinhos S. Miller J. A. (2013). Coexpression networks implicate human midfetal deep cortical projection neurons in the pathogenesis of autism. Cell, 155(5), 997–1007. 24267886 10.1016/j.cell.2013.10.020 PMC3995413

[bibr61-1759091419854627] Xu P. Xu H. Tang X. Xu L. Wang Y. Guo L. Yang Z. Xing Y. Wu Y. Warner M. (2014). Liver X receptor β is essential for the differentiation of radial glial cells to oligodendrocytes in the dorsal cortex. Mol Psych, 19(8), 947. 10.1038/mp.2014.60 24934178

[bibr62-1759091419854627] Yu Q. Ouyang A. Chalak L. Jeon T. Chia J. Mishra V. Sivarajan M. Jackson G. Rollins N. Liu S. (2016). Structural development of human fetal and preterm brain cortical plate based on population-averaged templates. Cereb Cortex, 26(11), 4381–4391. 26405055 10.1093/cercor/bhv201 PMC5066822

[bibr63-1759091419854627] Zhang B. Horvath S. (2005). A general framework for weighted gene co-expression network analysis. Stat Appl Genet Mol Biol, 4(1), 1–45. 10.2202/1544-6115.1128 16646834

[bibr64-1759091419854627] Ziats M. N. Rennert O. M. (2014). Identification of differentially expressed microRNAs across the developing human brain. Mol Psych, 19(7), 848. 10.1038/mp.2013.93 PMC3840150 23917947

