# Supplementary material for: Identification of the Molecular Events Involved in the Development of Prefrontal Cortex Through the Analysis of RNA-Seq Data From BrainSpan
Source: ASN Neuro. 2019 Jun 18;11:1759091419854627. doi: 10.1177/1759091419854627 (PMC6582306; doi:10.1177/1759091419854627)

Supplemental file S4. correlation between the expression level of three transcription factors (*TBP*, *RNase-H1* and *SPARCA2*) (y-axes) and global transcription rate (x-axes). Asterisks above the regression line denote the statistically significant at the level of *P*-value < 0.05.

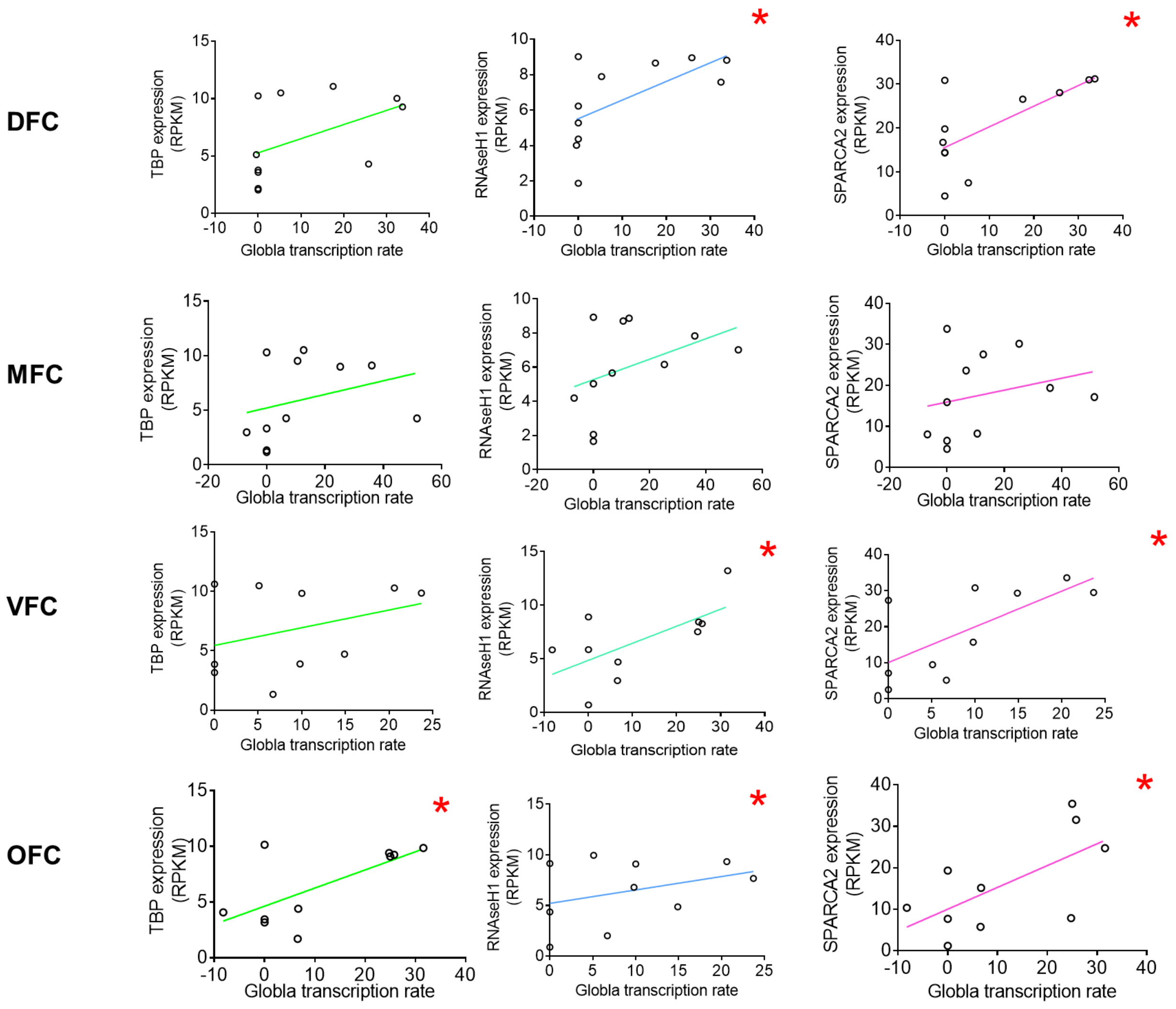

Supplement: Supplemental Material4 - Supplemental material for Identification of the Molecular Events Involved in the Development of Prefrontal Cortex Through the Analysis of RNA-Seq Data From BrainSpan [file Supplemental_Material4.pdf]
