# Supplementary material for: Identification of the Molecular Events Involved in the Development of Prefrontal Cortex Through the Analysis of RNA-Seq Data From BrainSpan
Source: ASN Neuro. 2019 Jun 18;11:1759091419854627. doi: 10.1177/1759091419854627 (PMC6582306; doi:10.1177/1759091419854627)

Supplemental file S5. Number of DEGs across developmental transitions of four different regions of prefrontal cortex after downsampling method on the RNA-seq data of the BrainSpan.

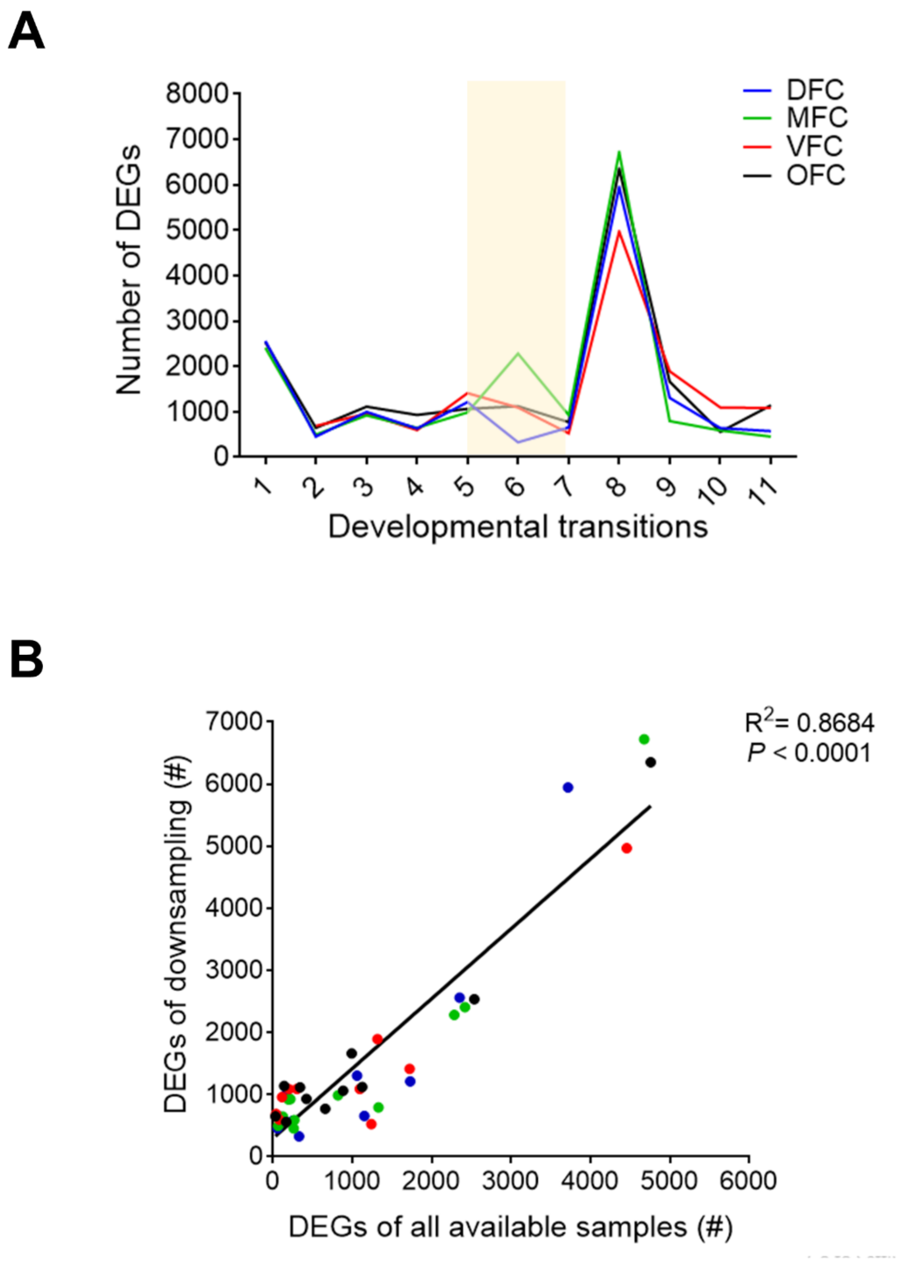

Supplement: Supplemental Material5 - Supplemental material for Identification of the Molecular Events Involved in the Development of Prefrontal Cortex Through the Analysis of RNA-Seq Data From BrainSpan [file Supplemental_Material5.pdf]
